# Computational prediction of diagnosis and feature selection on mesothelioma patient health records

**DOI:** 10.1371/journal.pone.0208737

**Published:** 2019-01-10

**Authors:** Davide Chicco, Cristina Rovelli

**Affiliations:** 1 Peter Munk Cardiac Centre, Toronto, Ontario, Canada; 2 Princess Margaret Cancer Centre, Toronto, Ontario, Canada; 3 Department of Molecular Genetics, University of Toronto, Toronto, Ontario, Canada; Universitat Politecnica de Catalunya, UNITED STATES

## Abstract

**Background:**

Mesothelioma is a lung cancer that kills thousands of people worldwide annually, especially those with exposure to asbestos. Diagnosis of mesothelioma in patients often requires time-consuming imaging techniques and biopsies. Machine learning can provide for a more effective, cheaper, and faster patient diagnosis and feature selection from clinical data in patient records.

**Methods and findings:**

We analyzed a dataset of health records of 324 patients having mesothelioma symptoms from Turkey. The patients had prior asbestos exposure and displayed symptoms consistent with mesothelioma. We compared probabilistic neural network, perceptron-based neural network, random forest, one rule, and decision tree classifiers to predict diagnosis of the patient records. We measured classifiers’ performance through standard confusion matrix scores such as Matthews correlation coefficient (MCC). Random forest outperformed all models tried, obtaining MCC = +0.37 on the complete imbalanced dataset and MCC = +0.64 on the under-sampled balanced dataset. We then employed random forest feature selection to identify the two most relevant dataset traits associated with mesothelioma: lung side and platelet count. These two risk factors resulted so predictive, that decision tree focusing on them achieved the second top accuracy on the complete dataset diagnosis prediction (MCC = +0.28), outperforming all other methods and even decision tree itself applied to all features.

**Conclusions:**

Our results show that machine learning can predict diagnoses of patients having mesothelioma symptoms with high accuracy, sensitivity, and specificity, in few minutes. Additionally, random forest can efficiently select the most important features of this clinical dataset (lung side and platelet count) in few seconds. The importance of pleural plaques in lung sides and blood platelets in mesothelioma diagnosis indicates that physicians should focus on these two features when reading records of patients with mesothelioma symptoms. Moreover, doctors can exploit our machinery to predict patient diagnosis when only lung side and platelet data are available.

## 1 Introduction

Mesothelioma is a major type of lung cancer. Incidence varies markedly by country [1, 2]. Between 2004 and 2008, 23,869 people in the Americas, 49,779 people in Europe, and 12,012 people in Asia died of mesothelioma [3].

Pleural mesothelioma makes up approximately 75% of all mesotheliomas, and affects the two membranes of the lung: the visceral pleura and parietal pleura. Other subtypes include pericardial mesothelioma, which develops in the membrane around the heart, the pericardium. In many cases, pericardial mesothelioma goes undiagnosed until autopsy [4]. Mesotheliomas are always malignant, but some patients with mesothelioma symptoms might have pleural plaques instead [5], without mesothelioma.

The most important symptoms for diagnosis include pain, dyspnoea (shortness of breath), cough, pain and dry cough, pleural effusion, chest pain, and shoulder pain. In more advanced stages, other symptoms can show up: weakness, fever, hoarseness, hypoxemia (low level of oxygen in the blood), dysphagia (difficulty swallowing), fever, night sweats, and weight loss [6, 7].

In contrast with symptoms, clinical features provide quantitative information to aid diagnosis. Existing models that forecast patient survival use clinical features such as histologic subtype, time since diagnosis, platelet count, hemoglobin, and disease stage [6]. In mesothelioma, occupational history generally can serve as a particularly informative feature, as it shows previous exposure to asbestos. Long workplace exposure to asbestos makes development of pleural mesothelioma extremely likely [8].

Mesothelioma diagnosis generally requires expensive imaging and laboratory medicine resources [9], such as X-rays, magnetic resonance imaging (MRI) and positron emission tomography (PET) scans, biopsies, and blood tests. Even if precise and efficient, the medical imaging machines are expensive and uncommon in remote regions. Medical tests like biopsies, in addition, are quite invasive and painful for patients.

To speed diagnosis and minimize the use of these tests, researchers have used machine learning methods to solve health informatics classification tasks [10]. Machine learning methods provide useful tools to classify, process, and analyze health records in minutes or seconds.

We analyzed a dataset of mesothelioma health records of 324 mesothelioma patients from the Diyarbakır region of southeast Turkey [11]. This area has endemic, natural asbestos fibers in the soil, mostly tremolite fibers, but also chrysotile fibers. This provides a unique dataset with a high incidence of mesothelioma within a population of highly asbestos-exposed individuals. From this dataset, we ascertained risk factors for mesothelioma. Each patient record in this dataset contains multiple clinical features and a diagnosis label. The diagnosis label has two categories: mesothelioma or non-mesothelioma. The “non-mesothelioma” patients have similar clinical features as those with mesothelioma, such as pleural plaques. Nonetheless, physicians did not diagnose these patients with mesothelioma.

It is often difficult to distinguish clinically between patients with mesothelioma with asbestos-exposed individuals who have pleural plaques and clinical features suggestive of mesothelioma, but who lack the disease. Asbestos exposure itself can lead to pleural plaque development, pleural effusions, and other radiologic changes that mimic mesothelioma.

Machine learning methods are well-established in scientific research for cancer predictive diagnosis [12–15]. The mesothelioma dataset used here comes from a previous effort to diagnose mesothelioma using a probabilistic neural network (PNN) [10]. Probabilistic neural networks have also been used to diagnose potential cancer patients [16–18], and to predict anti-HIV drugs [19]. To replicate the approach used by the original dataset authors [10], we started this study by reimplementing a probabilistic neural network [20], and then compared this algorithm with other machine learning models such as artificial neural networks [21, 22], random forests [23, 24], decision trees [25], and one rule [26].

We chose these methods because they are particularly appropriate for the dataset we analyze, and because they have proven successful and suitable in solving similar health informatics problems in the past [27, 28]. Artificial neural networks, for example, have been used to predict the sequence specificity of DNA-binding and RNA-binding proteins [29], and classify micrographs of breast cancer [30]. Random forests, also, have seen extensive use in bioinformatics and biomedical informatics contexts [31–33], such as for the classification of gene expression microarray data [34]. Additionally, researchers have used random forests to classify other cancer types, including renal cell carcinoma data [35], and lung cancer data [36]. Even if machine learning experts often suggest to start with a simple machine learning algorithm [37], such as logistic regression, we decided to avoid this method because it can be imprecise when applied to data having highly correlated features [38]. The mesothelioma dataset, in fact, contains highly correlated data features, generated from the same clinical tests. In addition to random forest’s use for classification, we also employed it for feature selection [31, 35, 39], to understand which patient traits and clinical features best predict mesothelioma.

Our findings and our methods can be useful for physicians and medical doctors, in several contexts. Our discoveries about the importance of lung side and platelet count in the dataset suggest physicians should focus on these two features, when reading the electronic health record of a patient. Additionally, physicians can take advantage of our method to predict if a patient is going to have mesothelioma or not, by inputing his/her clinical profile to our software.

## 2 Dataset

The dataset consists in real electronic health records of 324 patients collected at the Dicle University Faculty of Medicine Hospital (Diyarbakir, southeastern Turkey), before October 2011 [10]. Of these 324 patients, 96 have mesothelioma, and 228 have do not have mesothelioma. Regarding dataset imbalance, the data contains 29.63% positive data instances (patients with mesothelioma), and 70.37% negative instances (patients without mesothelioma).

We represent the dataset as a table of 324 rows, each row corresponding to one patient with potential mesothelioma symptoms. Each row has 35 columns, representing the observed features for that patient (Table 1). One of the features is the diagnosis label, “class of diagnosis”. This feature states whether the patient actually has mesothelioma (1, “mesothelioma” label), or or not (0, “non-mesothelioma” label).

**Table 1:** Dataset features with ranges and measurement units. We removed “diagnosis method” from the classification and feature selection phases, because it has the same values of “class of diagnosis” target we predict. We changed some feature names to add clarity: “blood lactic dehydrogenise (LDH)” into “lactate dehydrogenase test”, “cell count (WBC)” into “white blood cells (WBC)”, “cytology” into “cytology exam of pleural fluid”, “hemoglobin (HGB)” into “hemoglobin normality test”, “keep side” into “lung side”, “pleural glucose” into “pleural fluid glucose”, and “white blood” into “pleural fluid WBC count”.

feature name	value range	measurement unit
ache on chest	0, 1	boolean
asbestos exposure	0, 1	boolean
cytology exam of pleural fluid	0, 1	boolean
dead or not	0, 1	boolean
diagnosis method	0, 1	boolean
dyspnoea	0, 1	boolean
hemoglobin normality test	0, 1	boolean
pleural effusion	0, 1	boolean
pleural level of acidity (pH)	0, 1	boolean
pleural thickness on tomography	0, 1	boolean
weakness	0, 1	boolean
city	[0, 8]	category
gender	0, 1	category
habit of cigarette	0, 1, 2, 3	category
lung side	0, 1, 2	category
performance status	0, 1	category
type of malignant mesothelioma	0, 1, 2	category
age	[19, 85]	years
duration of asbestos exposure	[0, 70]	years
duration of symptoms	[0.5, 52]	years
albumin	[1.5, 6.9]	g/dL (grams per deciliter)
alkaline phosphatise (ALP)	[41, 489]	IU/L (international units per liter)
C-reactive protein (CRP)	[11, 103]	mg/L (milligrams per liter)
lactate dehydrogenase test (LDH)	[55, 1128]	IU/L (international units per liter)
glucose	[60, 421]	mg/dL (milligrams per deciliter)
platelet count (PLT)	[111, 3335]	kilo platelets per mcL (microliter)
pleural albumin	[0, 4.4]	g/dL (grams per deciliter)
pleural fluid WBC count	[742, 21500]	cells per microliter (mcL)
pleural fluid glucose	[2, 151]	mg/dL (milligrams per deciliter)
pleural lactic dehydrogenase	[110, 7541]	IU/L (international units per liter)
pleural protein	[0, 6.7]	g/L (grams per liter)
sedimentation rate	[7, 129]	mm/hr (millimeters per hour)
total protein	[3.1, 8.5]	g/dL (grams per deciliter)
white blood cells (WBC)	[4, 22]	cells per mcL (microliter)

The dataset curators published the first analysis of this dataset in October 2011 [10], and subsequently released the dataset publically on the University of California Irvine Machine Learning Repository in January 2016 [40]. Beyond the data origin, feature names, and their values, Er et al. [10] provided no other details about the dataset. We describe the features in more details here (Tables 1 and 2; S1, S2, S3, S4 and S5 Figs). The “diagnosis method” feature has identical values to “class of diagnosis” and we therefore removed it for classification and feature selection purposes. Of the 33 remaining features, 10 features are boolean, 14 are real values, 3 are time values, and 6 are categorical. We describe the features in depth (Tables 1 and 2, Supplementary Information) and confirmed our interpretation with the dataset curators (Orhan Er, personal communication).

**Table 2:** Meaning of each feature of the dataset. We reported a detailed description of each feature in the Supplementary Information.

feature name	meaning
ache on chest	presence or absence of pain in the chest area
asbestos exposure	if a patient has been exposed to asbestos during life
cytology exam of pleural fluid	test to detect cancer cells and certain other cells in the area that surrounds the lung
dead or not	if a patient is still alive
diagnosis method	if the patient has had a mesothelioma diagnosed by a common diagnosis method
dyspnoea	shortness of breath
hemoglobin normality test	test that measures how much hemoglobin is in blood
pleural effusion	presence of effusion, common symptom that can inhibit the normal function of the organ
pleural level of acidity (pH)	if the pleural fluid pH is lower than the normal pleural fluid pH, that it’s neutral
pleural thickness of thickness	any form of thickening involving either the parietal or visceral pleura
weakness	lack of strength
city	place of provenance of the patients
gender	female or male
habit of cigarette	four categories for the habit of smoking
lung side	the side of the lungs which is experiencing pleural plaques or mesothelioma traces
performance status	patient’s ability to perform normal tasks
type of malignant mesothelioma	mesothelioma stage to which the symptoms seem to belong, according to the TNM Classification of Malignant Tumors
age	the age of the patients
duration of asbestos exposure	how long has been the environmental exposure to asbestos
duration of symptoms	the time period, in years, in which the patients show symptoms
albumin	level of blood albumin
alkaline phosphatase (ALP)	test used to help detect liver disease or bone disorders
C-reactive protein (CRP)	acute phase reactant, significantly elevated in patients with pleural mesothelioma (MPM)
glucose	test which measures the amount of glucose in a sample of blood
lactate dehydrogenase test (LDH)	protein that helps produce energy in the body
platelet count (PLT)	test to measure how many platelets patients have in the blood
pleural albumin	level of albumin in the pleural fluid
pleural fluid WBC count	the count of leukocytes in the pleural fluid
pleural fluid glucose	low level can be linked to infection or malignancy
pleural lactic dehydrogenase	its levels indicates if the fluid is exudate or transudate
pleural protein	pleural effusions are classified as transudates or exudates on the basis of the fluid protein level
sedimentation rate	test to measure how quickly erythrocytes settle in a test tube in one hour
total protein	biochemical test for measuring the total amount of protein in serum
white blood cells (WBC)	test measures the number and quality of white blood cells

It is also worth noticing that the dataset is well structured and complete, and contains no missing or ambiguous values. The dataset contains only real patients’ data, and no simulations. The completeness of the dataset is a rare quality in electronic health record (EHR) collections, and allows us to make a more precise and accurate analysis than other cases where some data values are missing (for example, [41]).

## 3 Methods

In the first part of the project, we used machine learning to perform a supervised binary prediction of the two possible patient diagnoses (mesothelioma or non-mesothelioma).

To this end, we took advantage of several models. We started with PNN, since it was the method applied by the original dataset authors [10], and we wanted to replicate their approach. To further investigate the effectiveness of artificial neural networks, we then used a perceptron-based neural network.

Afterwards, we decided to move to tree-like graph models (decision trees, random forest, and one rule), because these methods are unmoved by statistical correlations between features, which are very common in electronic health record datasets. Clinical data, in fact, contain features that have strong relationships between each other, since each aspect of the health of a patient is deeply related to her/his other health aspects, at any level. Tree-like graph models usually are minimally affected by feature correlations, and therefore they can be efficient and robust when applied to patient clinical datasets, as in this case.

In the second part of the project, we investigated the most relevant features associated with mesothelioma. For this purpose, we decided to use random forest feature selection because this method achieved the best results in predicting the diagnosis (Results). We also wanted to take advantage of its ensemble learning approach and importance rates (accuracy decrease and Gini impurity decrease), which let us understand the importance of each feature both statistically and informatively. We decided to avoid employing the other methods for this feature selection phase because they do not provide an informative content such as the Gini impurity decrease [39]. Additionally, even if our feature selection results might be biased towards random forest, we preferred this technique because *bootstrap aggregating* [42] makes ensemble learning methods more robust than neural networks, decision trees, and association rule learning algorithms, regarding feature selection [36, 43, 44].

### 3.1 Probabilistic neural network

**Fig 1:**
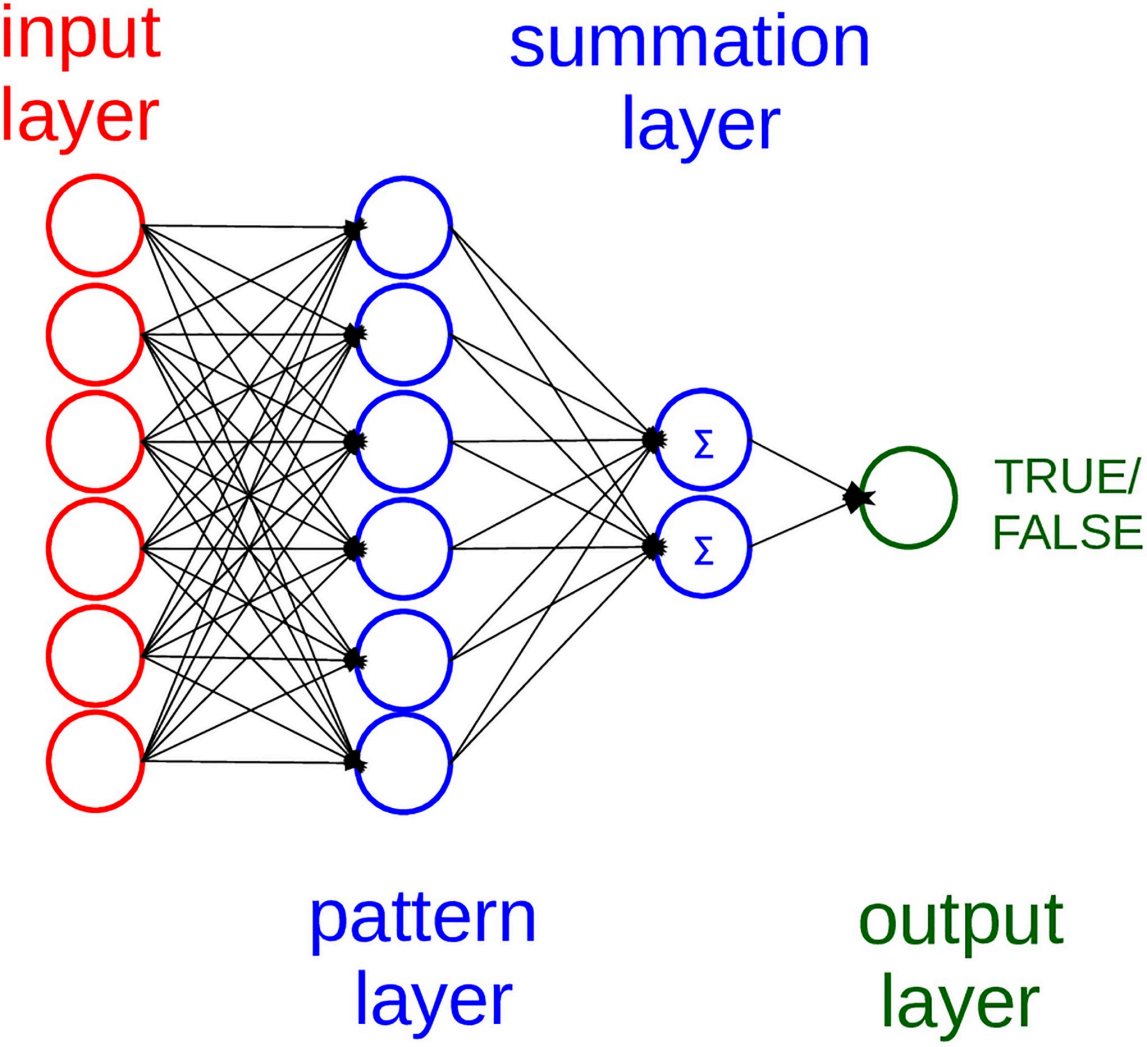
Architecture of the probabilistic neural network. In our model, there are 33 neurons in the input layer, 33 neurons in the pattern layer, and 2 neurons in the summation layer.

The probabilistic neural network is an artificial neural network algorithm based upon a Bayesian statistical network and a Fisher kernel discriminant analysis model [20] (Fig 1).

**Fig 2:**
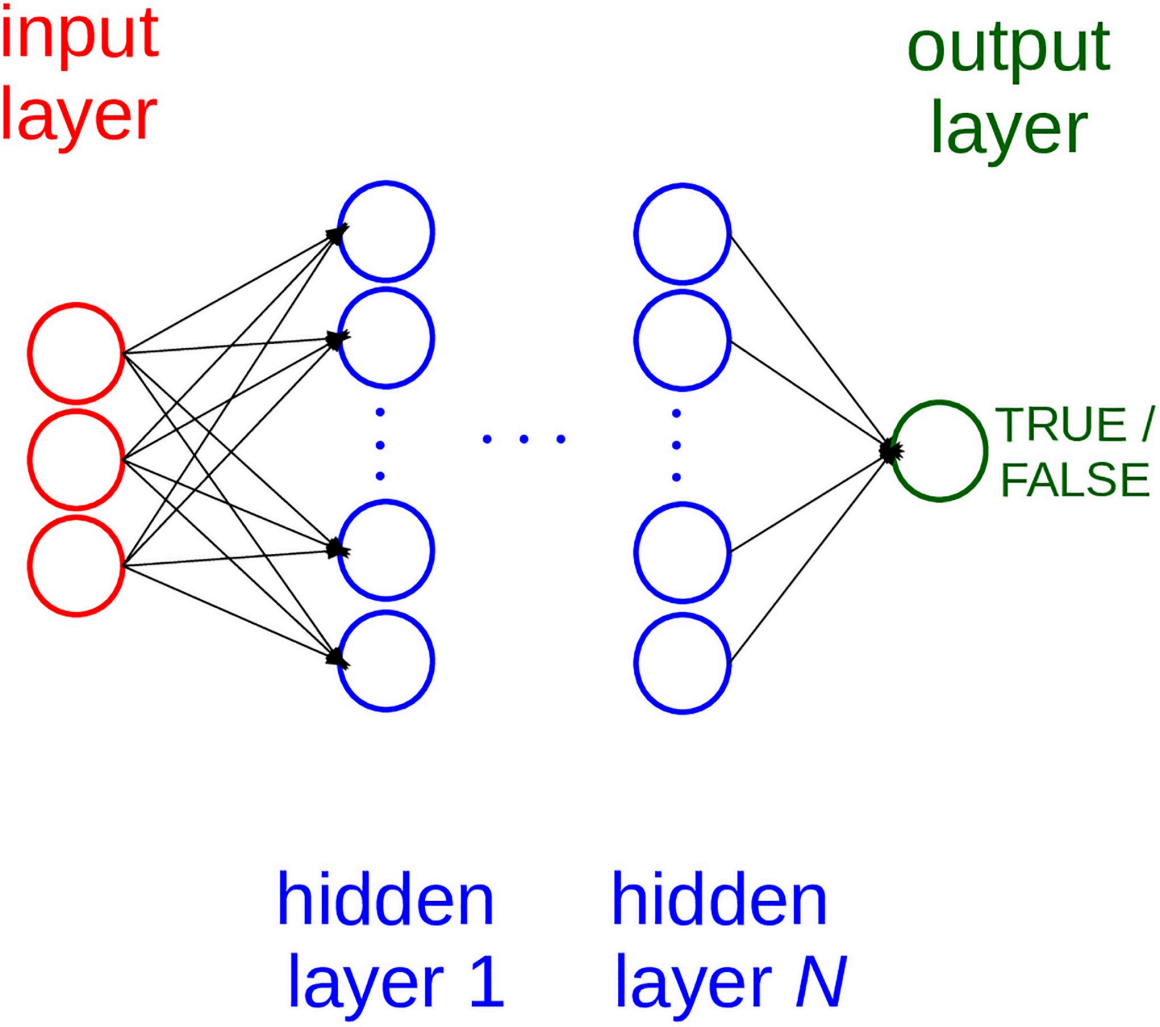
Architecture of a multi-layer perceptron-based neural network. In our model, the input layer neurons are 33. We found different optimized numbers of hidden layers and hidden units, for each program execution. The top architecture among the ten executions had 20 hidden units and 1 hidden layer.

A typical artificial neural network contains one input layer, several hidden layers, and an output layer. Each neuron of the input layer contains a value that propagates to the first hidden layer neurons. In feed-forward neural networks (such as probabilistic neural networks and perceptrons), each hidden layer neuron reads the input layer values, multiply them by its weights, sums the temporary results up, applies an activation function, and propagates its result to the next layer of neurons. A multi-layer feed-forward perceptron is a typical artificial neural network, made of one input layer, several hidden layers, and an output layer (Fig 2).

Unlike the classical multi-layer perceptron [45], which has a back-propagation method that updates the weights of the neurons at each iteration, the probabilistic neural network computes as output values as probability of class membership. A probabilistic neural network consists of an input layer, a pattern layer, a summation layer, and an output layer. The input layer reads the input values, while the pattern layer computes the radial distance between the values of each pair of input neurons, through a Gaussian function. In the summation layer, the neural network sums all the values output by the previous layer, generating probability values that estimate the likelihood of class membership in the output layer. For a supervised binary classification, the method assigns each value to the most likely boolean category it can belong, true or false (Fig 1).

This particular artificial neural network is a lazy learning model, meaning that it does include an iterative training procedure. When using a probabilistic neural network, we do not train the neurons’ weights, but rather assign values to them (Methods).

Following the strategy initially adopted by the dataset curators [10], we implemented and tested a probabilistic neural network. We set the model Gaussian function to have a standard deviation value of 0.1.

We read the 33 feature values of each patient in the input layer, and we processed their values in the hidden layer. Then, in the output layer, we estimated whether the patient belongs in the mesothelioma or non-mesothelioma diagnosis class. We used 5-fold cross-validation. In each cross-validation fold, we trained on a randomly chosen 80% of the patients, and test on the remaining 20% of the patients. The algorithm finally states if each patient profile is more likely to to belong to the mesothelioma class, or to the non-mesothelioma class.

For our tests, we split the dataset into training set and test set, as made by the dataset curators [10]. We trained our model on the training set, and then applied the trained model to the test set. Best practices in machine learning suggest to split the original dataset into three independent subsets (training set, validation set, and test set) [37], but we decided to use only two-subset split to reproduce the probabilistic neural network used [10]. We then split the dataset into training set, validation set, and test set for the perceptron; we then trained each perceptron model on the training set, evaluated it with different hyper-parameters on the validation set, and finally applied the top performing model to the test set.

### 3.2 Perceptron-based neural network

The main difference between a perceptron and a probabilistic neural network comes from back-propagation. In the perceptron, once the values propagate the neural network and reach the output layer, the neural network computes the mean square error between the predicted values and the gold-standard values. Afterwards, the algorithm sends this error measure back to neurons of each hidden layer, through a technique called back-propagation [46], and they update their weights accordingly.

We read the 33 input values of each patient profile in the input layer, then learned a hidden representation of the profile, and finally translated it into a single real-valued score in the output layer.

We set a confusion matrix threshold *τ* to 0.5. During testing, we scaled all the values outputted by the neural network through the *z* = (*x* + 1)/2 formula, where *x* is the output of the perceptron, and *z* is the actual value used in the confusion matrix. If the prediction generated a score greater than the likelihood threshold *τ*, we assigned the patient to the non-mesothelioma class. Otherwise, we assign the patient to the mesothelioma class.

Our multi-layer perceptron used a learning rate of 0.01, and 200 iterations in training. We computed the confusion matrix with the likelihood threshold *τ* = 0.5. We normalized the input data by column, by scaling every value into the [0; 1] interval, before the application of the perceptron.

We optimized the hyper-parameters (number of hidden layers and number of hidden units) through a grid search, by testing several possible values (hidden layers = [1, 2, 3] and hidden units = [5, 10, 20, 25, 75, 100]). We randomly separated the original dataset into three independent subsets: training set (60% patients, randomly selected), validation set (other 20% patients, randomly selected), and a test set (the remaining 20% patients).

During optimization, for each hyper-parameter configuration, we trained the perceptron on the training set and tested in on the validation set, by computing the Matthews correlation coefficient (MCC) [37, 47]. At the end of the optimization phase, we selected the model which led to the highest MCC score, and applied it to the test set.

Our optimization tests led to different optimized number of hidden units and hidden layers each time, and obtained the top prediction results (MCC = +0.27) with 1 hidden layer and 20 hidden units. In our neural network, we used the sigmoid as activation function.

### 3.3 Random forest and decision trees

Random forest build upon decision tree learning, in which a set of predictive decision trees maps each input item into an output category, by processing it through its tree leaves [23, 24].

A decision tree is a classification model in which every node is a decision function, and the node child represents every potential choice from that decision. The tree applies the decision function of each node repeatedly to the input, and then associates the data sample to the corresponding child. Afterwards, the child also applies its decision function to the input, and associates it to one of its child nodes, and so on. The algorithm repeats this procedure until it reaches the end of the tree.

Random forest is an ensemble learning method: it generates multiple classifiers and then aggregates their results. During training, random forest applies a bootstrap aggregating (bagging) method to its trees. It selects random subsets from the input dataset, and applies a decision tree to each of them. To select the final classification outcome, it selects the outcome produced by the majority of the trees, much like a voting system.

**Fig 3:**
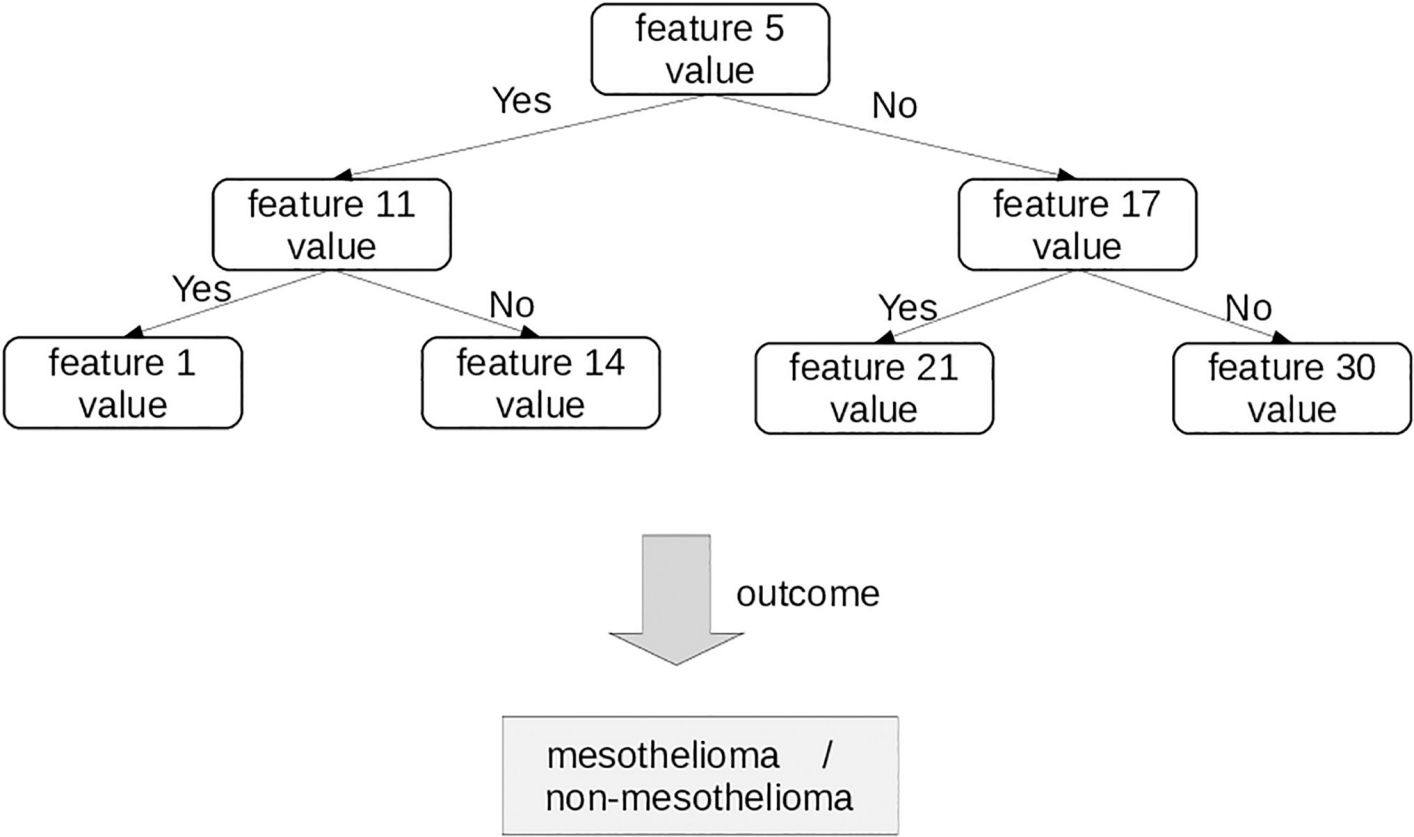
Decision tree. An example of decision tree, which can classify each patient as healthy (non-mesothelioma) or unhealthy (mesothelioma). Random forest generates a set of predictive decision trees.

The algorithm creates several random decision trees, in which every node corresponds to a feature, randomly selected (Fig 3). The algorithm applies a decision function to each patient profile. For example, for each boolean feature, the node function is “Is the value true?”. By applying decision functions repeatedly at each node, the algorithm finally classifies a whole data sample as true or false (mesothelioma or non-mesothelioma in our case). In the end, the random forest outputs the outcome class corresponding to the majority of the outcome classes of the random decision trees (Fig 3), and classifies it as true or false. We trained our random forest classifier on randomly selected 80% data instances and tested on the remaining 20%. In our random forest implementation, we generated 500 trees and tried 11 variables at each node split. Differently from the numbers of hidden units and hidden layers of the previously described perceptron-based neural network, we did not run an optimization procedure for the number of generated trees. Increasing the number of trees, in fact, does not improve the performance of random forest, if the the number of trees is sufficiently larger than the number of features [48, 49].

After the diagnosis classification phase, we decided to investigate the most important features of the dataset. We again chose to use random forest for this scope, because this method provides both a statistical outcome (accuracy decrease) and a content-informative outcome of the importance of each feature (Gini node impurity). All the other methods previously used for classification in this project (PNN, perceptron, and one rule) do not produce this twofold outcome.

To rank the importance of each feature, we applied the random forest algorithm to the dataset 33 times. Each time, we removed one feature of the 33 and then computed the accuracy (Eq 1) and the Gini node impurity [39] of the prediction during the decision tree training.

In a confusion matrix, where FP: false positives; FN: false negatives; TP: true positives; TN: true negatives, the accuracy formula is the following:

$$\begin{array}{c} \text{accuracy}=\frac{\text{TP}+\text{TN}}{\text{TP}+\text{FN}+\text{TN}+\text{FP}}\\ \left(\text{accuracy}:\text{worst}\;\text{value}=0;\text{best}\;\text{value}=1\right) \end{array}$$ (1)

Then, we measured the accuracy and the Gini node impurity decrease between the random forest with all features and the random forest with one feature removed. The top importance features are the ones whose difference in the accuracy and in the Gini node purity is higher, because its absence shows the largest change in the prediction of the diagnosis.

Even if accuracy is less informative than MCC on imbalanced dataset [37], we decided to stick with this score in the feature selection phase to be consistent with the original random forest model introduced by Breiman [23].

We applied the random forest algorithm to rank the features based upon their importance. We measured feature importance with two statistical rates: the proportion of the decrease of the mean square error (MSE, Eq 2) when each feature is missing from the dataset, measured against the 0 or 1 target value (Results), and the tree node Gini impurity (Results).

$$\begin{array}{c} \begin{array}{c} \text{MSE}\left(x,y\right)=||x-y|{|}^{2} \end{array}\\ \left(\text{where}\;x\;\text{is}\;\text{predicted}\;\text{score},\text{and}\;y\;\text{is}\;\text{the}\;\text{corresponding}\;\text{ground}\;\text{truth}\;\text{target}\right) \end{array}$$ (2)

We computed the percentage of the decrease of the mean square error in the following way. The random forest algorithm computed the accuracy *α*_all_ of the prediction of the targets by using a decision tree which takes advantage of all the features. Then, the random forest algorithm calculated the accuracy *α*_i_ of the prediction of the targets by using a decision tree which takes advantage of all the features, except the i_*th*_ feature.

Afterwards, for each feature *i*, it computed the percentage mean square error between *α*_all_ and *α*_i_, and assigned it to the *i*_*th*_ feature as its percentage of the decrease of the mean square error (Results).

The random forest algorithm computes the impurity of each tree node measured by the Gini index in the following way. For each decision tree split, the method calculated the decrease of Gini index impurity between the node before the split and the node after the split [39].

The larger the impurity decrease after a specific split, the more informative is the feature related to that split [50]. The algorithm summed over all the splits for that feature, over all the trees, and generates its final value (Results).

Feature selection measures the importance of each dataset feature through the accuracy decrease and the Gini impurity decrease. We can consider the accuracy decrease percentage of the mean square error as an importance measure in a statistical sense, and the tree node impurity as an importance measure in an informative content sense.

We applied the random forest feature selection algorithm on all the dataset, and computed the accuracy decrease and the Gini purity decrease for each feature (Results). Here there is no need to split the dataset into training set and test set, because random forest feature selection uses a technique called bagging (or bootstrap aggregation), which generates multiple data subsets by sampling with replacement from the full dataset [42].

We took the accuracy decrease ranking and the Gini impurity ranking, and created a merged ranking by using Borda’s method [51]. For each feature *f*, we sum its position in the first list *p*_1_(*f*) to its position in the second list *p*_2_(*f*), and save this value in the ranking score variable *score*_*f*_.

We then sorted all the features from the one having the lowest *score*_*f*_ value to the one having the highest score value (Results).

### 3.4 One rule

For a baseline comparison, we also implemented and applied the one rule algorithm [26]. Considered one of the simplest machine learning methods existing, one rule is based upon association rules, which involve just one data feature value in each rule condition. In the one rule application, we used randomly selected 80% of the data instances for training, and the remaining 20% for testing (Results).

### 3.5 Prediction using only two selected features

Since the random forest feature selection highlighted “lung side” and “platelet count (PLT)” as the most relevant features in the dataset (Results), we used a decision tree to predict mesothelioma diagnoses based solely upon these two features (Fig 3). We applied classification and regression tree (CART) [25] to the dataset made only of lung side and platelet count. In the dataset table, we kept only the “lung side” and “platelet count (PLT)” columns, we removed all the other columns (features), and then we applied the CART method. We avoided using random forest in this case because there are only two features: as we described earlier, random forest creates decisions trees based on random combinations of feature subsets, and there would be no possible subset combinations on a dataset containing only two features.

We decided to employ decision tree in this phase because lung side and platelet count were identified as the two most important features by random forest, and random forest is based upon decision trees [23]. If we used another method such as perceptron-based neural network at this stage, it could potentially disagree with random forest on which features are the most important, and therefore generate inconsistent diagnosis prediction results.

Moreover, a methodological advantage of decision tree is that its operating principles and results are easy to understand and interpret [52, 53]. In a scenario where a biomedical doctor has to figure out if a patient had mesothelioma by just looking at the values for lung side and platelet count in the medical record, he/she could diagram all the decision tree steps and understand the reasons beyond the outcome generated. This information would be pivotal for the doctor’s decision making, and would help him/her better interpret the patient’s situation [54, 55]. On the contrary, understanding the operating principles beyond more complex machine learning methods (such as the neural networks used in this study) would be difficult, or even impossible, in a health decision making context [56]. Therefore, to make a critical decision about the therapy for a patient, a biomedical doctor would trust an explainable decision tree more than a black box neural network.

To verify that the predictive power of the lung side and platelet count was valid not only for decision trees, we also applied one rule to this dataset made by only the two selected features (S4 Table).

We used the 80% randomly selected patient profiles for training and the remaining 20% profiles for testing (Results).

### 3.6 Prediction measurement and dataset split

To state the effectiveness of our prediction methods, we used Matthews correlation coefficient (MCC, Eq 3), accuracy (Eq 1), *F*_1_ score (Eq 4), sensitivity (true positive rate, Eq 5), and specificity (true negative rate, Eq 6) rates.

$$\begin{array}{c} \begin{array}{c} \text{MCC}=\frac{\text{TP}\cdot \text{TN}-\text{FP}\cdot \text{FN}}{\sqrt{\left(\text{TP}+\text{FP}\right)\cdot \left(\text{TP}+\text{FN}\right)\cdot \left(\text{TN}+\text{FP}\right)\cdot \left(\text{TN}+\text{FN}\right)}} \end{array}\\ \left(\text{MCC}:\text{worst}\;\text{value}=-1;\text{best}\;\text{value}=+1\right) \end{array}$$ (3)

$$\begin{array}{c} \begin{array}{c} {\text{F}}_{1}\;\text{score}=\frac{2\cdot \text{TP}}{2\cdot \text{TP}+\text{FN}+\text{FP}} \end{array}\\ \left({\text{F}}_{1}\;\text{score}:\text{worst}\;\text{value}=0;\text{best}\;\text{value}=1\right) \end{array}$$ (4)

$$\begin{array}{c} \begin{array}{c} \text{sensitivity}=\frac{\text{TP}}{\text{TP}+\text{FN}} \end{array}\\ \left(\text{sensitivity}:\text{worst}\;\text{value}=0;\text{best}\;\text{value}=1\right) \end{array}$$ (5)

$$\begin{array}{c} \begin{array}{c} \text{specificity}=\frac{\text{TN}}{\text{TN}+\text{FP}} \end{array}\\ \left(\text{specificity}:\text{worst}\;\text{value}=0;\text{best}\;\text{value}=1\right) \end{array}$$ (6)

We optimize and evaluate our methods by using the MCC because it weights each class of the confusion matrix, in proportion to the number of positive elements and negative elements in both the gold standard and the prediction [47]. Sensitivity (Eq 5) generates the rate of the correctly predicted true positives on the total positive data instances, while specificity (Eq 6) produces the rate of the correctly classified true negatives on the total tally of false data instances. Accuracy (Eq 1) measures the proportion of the correct predictions (true positives plus true negatives) on the total data instances, while the F_1_ score (Eq 4) reports the is the harmonic mean of precision and sensitivity.

As described earlier, we used different strategies for the dataset split, in accordance to the need of optimizing hyper-parameters or not, for each method. Since our probabilistic neural networks, one rule, random forest, and decision trees have no hyper-parameter to optimize, we split the dataset into training set (80% of the data instances, randomly selected) and test test (the remaining 20% data instances) for all the analyses [37].

For the perceptron-based neural network, instead, we ran an optimization procedure to find the best number of hidden units and number of hidden layers. To do so, we split the dataset into training set (60% data instances, randomly selected), validation set (20% of the remaining data instances, randomly selected), and test set (the remaining 20% data instances). We trained each architecture model on the training set, and evaluated it on the validation set. At the end of the optimization phase, we selected the model which obtained the highest prediction score (MCC) on the validation set, and finally applied to the test set.

We later report the results related to the test sets (Results). Each test set used by each method in this project was completely independent from training set and validation set, and has no element in common with them. Each test set employed for each method execution contains 20% of the dataset: 65 randomly selected patients for the complete imbalance dataset tests, and 39 randomly selected patients for the under-sampled balanced dataset tests.

To make an even more precise comparison of the classifiers we employed, we recognize that it would have been ideal to initially put aside a *held-out* data subset as an additional final test set [57], then apply all the optimized trained methods on this *held-out* subset, and finally compare the results obtained by each method (similarly to what happens in the DREAM Challenges [58, 59]). Unfortunately, because of the small size of the dataset analyzed in this study (324 patients), we could not take advantage of this strategy, otherwise we would have not had enough data instances to properly train the models. Our results attained by randomly selecting and shuffling the data instances for each test set, however, confirmed the generalisability of our methods.

### 3.7 Regression analysis

In addition to the neural network, random forest, one rule, and decision tree prediction and random forest feature selection approaches, we also applied a traditional regression analysis to the dataset. We compared of clinical, radiographic, demographic, and laboratory characteristics between the mesothelioma patients and those who do not have cancer (non-mesothelioma) but who are asbestos exposed by Wilcoxon rank sum tests for continuous variables [60], and Fisher exact tests for categorical variables [61]. We applied univariate logistic regression models to assess the effect of each individual factor or characteristics on diagnosis, reporting estimated odds ratios (OR) and 95% confidence intervals (CI) after applying two-sided statistical tests. A multivariate logistic regression model included all variables with a threshold alpha set at 0.10 or lower, followed by backwards selection of variables with a threshold set at alpha of 0.05 or lower.

### 3.8 Execution details

On a Dell Latitude 3540 computer with a Intel Core i3-4030U CPU 1.90 GHz processor, with 3.7 GB of random-access memory (RAM), and running a Linux CentOS 7 operating system, the execution of the probabilistic neural network on Python 3.5 with NeuPy [62] execution takes around 1 second, while the execution of the perceptron-based neural network on Torch 7 with the nn and optim packages [63] execution takes around 2 minutes and 30 seconds. The perceptron prediction takes longer because of the optimization phase, which lacks for the other algorithms. We applied random forest through the R randomForest package, and its execution lasted around 1 second, both for classification and feature selection. We applied one rule through the R OneR package [64] and the CART decision tree through the R rpart package [65], and their execution lasted around 1 second, too.

## 4 Results

### 4.1 Predictions of patients diagnosis on the complete imbalanced dataset

Er et al. [10] reported top prediction accuracy of 0.98, but, upon investigation, we noticed that one of their input feature (“diagnosis method”) duplicated the target diagnosis class. This input feature makes it trivially easy to obtain perfect and almost perfect prediction accuracy, but it is unlikely to exist in a real-world setting. Therefore, we excluded this feature from our analysis.

We generated prediction results through probabilistic neural network, perceptron-based neural network, one rule, decision tree, and random forest classifier applied to the all the features and to all the data instances, and through decision tree applied to the two top selected features and to all the data instances (Table 3).

**Table 3:** Results of the computational predictions of patient diagnosis on the complete dataset. Matthews correlation coefficient (MCC): Eq 3. Accuracy: Eq 1. F_1_ score: Eq 4. Sensitivity (true positive rate): Eq 5. Specificity (true negative rate): Eq 6. The scores are the medians of the results’ ten separate program executions. We report the results of the application of the methods on all the dataset features, plus the results of the decision tree only to the two selected features: the row entitled “Decision tree (applied only to lung side & platelet count)”. Dataset imbalance: 29.63% positive data instances (all the 96 mesothelioma patients), and 70.37% negative data instances (all the 228 non-mesothelioma patients).

method	MCC	accuracy	*F*_1_ score	sensitivity	specificity
Random forest classifier	**+0.37**	0.75	0.39	0.28	0.97
Decision tree (applied only to lung side & platelet count)	**+0.28**	0.76	0.37	0.28	0.95
One rule	**+0.27**	0.74	0.29	0.17	0.97
Decision tree	**+0.19**	0.69	0.39	0.39	0.80
Perceptron	**+0.11**	0.52	0.47	0.66	0.42
Probabilistic neural network	**+0.03**	0.57	0.32	0.32	0.71

Our probabilistic neural network achieved the lowest prediction score among the methods we tried, obtaining a result similar to a random prediction (MCC = +0.03, Table 3). This method showed flaws in predicting true positives (sensitivity = 0.32) but did sufficiently well on predicting true negatives (specificity = 0.71).

The other artificial neural network we used, the multi-layer perceptron, and decision tree attained a low general scores: MCC = +0.11 and MCC = +0.19, respectively (Table 3). This deep learning model obtained a low prediction score on the true negatives (specificity = 0.42) but very good prediction score on the true positives (sensitivity = 0.66, (Table 3). The CART decision tree achieved specificity (0.80) but low sensitivity (0.39).

Regarding tree like graph models, one rule (MCC = +0.27) achieved very low results on the sensitivity (0.17) but almost perfect predictions on the specificity (0.97). Random forest outperformed all the other methods, achieving MCC = +0.37, with a low true positive rate (sensitivity = 0.28) and an almost perfect true negative rate (specificity = 0.97).

We also took advantage of the feature selection discoveries, and applied a CART decision tree [25] only to the “lung side” and “platelet count (PLT)” patients values. The prediction results showed an MCC of +0.28, higher than the results obtained with one rule (MCC = +0.27), of multi-layer perceptron (MCC = +0.11), of the probabilistic neural network (MCC = +0.03), and even of decision tree itself (MCC = +0.19) applied to the complete dataset (Table 3). These results confirmed that “lung side” and “platelet count (PLT)” are the most relevant features of the dataset in our analysis, and are alone sufficient to run a reliable computational prediction of the patients’ true negative outcomes. To prove these results on the selected two features are unbiased towards the CART decision tree, we applied one rule to the same dataset and obtained similar results, even if slightly lower (MCC = +0.27, S4 Table).

Generally, random forest outperformed all the other methods on the MCC and true negative rate (specificity), but was outperformed by perceptron and probabilistic neural network on the true positive rate (sensitivity). The decision tree applied to the two features obtained the top accuracy, while the multi-layer perceptron was the only algorithm which achieved high prediction results for true positive patients (sensitivity = 0.66), while all the other methods obtain sensitivity scores lower than 0.5, so cannot be considered reliable in detecting true positive patients (Table 3). The multi-layer perceptron attained a true negative rate (specificity = 0.42) lower than all the other methods (Table 3).

Sensitivity and specificity results show that all the methods except the multi-layer perceptron had better capability in predicting true negatives than true positives (Table 3). We believe these results are caused by an imbalanced ratio (29.63% positive data instances, and 70.37% negative instances) of the dataset. Since the models see more negative elements during training, they are better at predicting negative data instances during testing. We therefore tacked the dataset imbalance problem with the under-sampling technique [66], and we show the results in the next section.

However, this inability to predict true positives does not regard the multi-layer neural network, which achieved a high true positive rate (sensitivity = 0.66) without any data imbalance handling strategy.

Even if correctly classifying patients with mesothelioma (sensitivity) and patients without mesothelioma (specificity) are both relevant tasks, we give more importance to the former, because it can identify the patients that need to be cured through a therapy, and possibly have their life saved by an early detection of mesothelioma. To this end, it is relevant to notice that the decision tree applied only to the lung side and platelet count features gained a higher sensitivity (0.28) than the random forest classifier and one rule (Table 3). Regarding true negatives, it is worth mentioning that random forest classifier and one rule obtained an almost perfect specificity score (0.97) that outperformed all the other models (Table 3).

It is relevant to notice that each of the five confusion matrix scores we listed (MCC, accuracy, F_1_ score, sensitivity, specificity, Table 3) generate different rankings of our methods, confirming the importance of comparing different rates and not focusing on a single one. As mentioned earlier, we optimized our methods based upon the Matthews correlation coefficient, because it is the only rate that considers all the four categories of the confusion matrix and the balance of the dataset.

For a complete comparison to the reported results of Er et al. [10], we also computed the predictions on the original dataset including the problematic “diagnosis method” feature (S3 Table). As expected, random forest achieved perfect MCC of +1.00, but such classifier would have limited utility in clinical settings.

### 4.2 Predictions of patients diagnosis on the under-sampled balanced dataset

As already mentioned, our methods applied to the complete dataset obtained generally good results on the true negative rate, and low results on the true positive rate (Table 3). The dataset imbalance is the cause of this inability to predict true positives. The dataset, in fact, contains 228 negative data instances, and just 96 positive data instances. During training, then, each model learns well how to recognize negative elements, but does not learn well how to identify positive elements.

There are many techniques to tackle this dataset imbalance problem: data class weighting [67], over-sampling [68], and under-sampling [66], for example. Here we decided to use under-sampling because this approach does not involve any manipulation or weight assignment to the data instances, making its application more realistically usable in clinical environments than other techniques.

We implemented under-sampling in the following way. The minority class in our dataset contains 96 elements (positive data instances), while majority class contains 228 elements (negative data instances). We created a balanced subset containing all the 96 positive data instances, and 96 negative data instances randomly selected from the majority class. The balanced subset created now contained 192 data instances, with 50% perfect balance. We then applied all the methods to this balanced subset (with the same dataset split and execution configurations of the previous tests) and recorded their results (Table 4).

**Table 4:** Results of the computational predictions of patient diagnosis, after under-sampling. Matthews correlation coefficient (MCC): Eq 3. Accuracy: Eq 1. F_1_ score: Eq 4. Sensitivity (true positive rate): Eq 5. Specificity (true negative rate): Eq 6. The scores are the medians of the results’ ten separate program executions, run with different subset content selected randomly for training set, validation set, and test set every time. We report the results of the application of the methods on all the dataset features, plus the results of the decision tree only to the two selected features: the row entitled “Decision tree (applied only to lung side & platelet count)”. Dataset balance: 50% positive data instances (all the 96 mesothelioma patients), and 50% negative data instances (96 non-mesothelioma patients, randomly selected). Perceptron: learning rate = 0.1.

method	MCC	accuracy	*F*_1_ score	sensitivity	specificity
Random forest classifier	**+0.64**	0.82	0.80	0.75	0.86
Decision tree	**+0.59**	0.79	0.77	0.72	0.82
Decision tree (applied only to lung side & platelet count)	**+0.41**	0.68	0.63	0.58	0.80
Perceptron	**+0.23**	0.62	0.71	0.95	0.20
One rule	**+0.15**	0.57	0.55	0.47	0.67
Probabilistic neural network	**+0.10**	0.53	0.50	0.50	0.58

Compared to the results obtained on all the dataset (Table 3), here all the methods achieved lower specificity and higher sensitivity (Table 4) correctly reflecting the change of ratio positive and negative data instances. After under-sampling, in fact, the percentage of negative data instances moved from 70.37% to 50%, while the percentage of positive data instances increased from 29.64% to 50%. These changes made all the methods able to learn a larger ratio of positive elements, and a smaller ratio of negative elements during training, and their consequences clearly influenced the results (Table 4).

Random forest outperformed again all the other methods (MCC = +0.64), by obtaining a high true positive rate (sensitivity = 0.75) and a very high true negative rate (specificity = 0.86). Among all the methods tried, random forest attained the best MCC, accuracy, F_1_ score, and true negative rate. Random forest, however, did not attain the top true positive rate, which was achieved again by the multi-layer perceptron neural network (sensitivity = 0.95). Perceptron-based neural network obtained the highest true positive rate both on the complete imbalanced dataset (Table 3) and on the under-sampled balanced dataset (Table 4).

Conversely from the results obtained on all the data instances (Table 3), decision tree applied on all the features of the under-sampled dataset achieved the second top performance among all the methods (MCC = +0.59) and outperformed decision tree itself applied just to the lung side and platelet count (MCC = +0.41). These results show that decision tree applied only to the two selected features works well if there are enough data instances to train and test the model; otherwise, more features lead to better prediction scores. On the complete imbalanced dataset, in fact, there are 324 patients for which the lung side and platelet count features are available. Here, instead, decision tree applied to the two-feature dataset made of just 192 patients did not have enough data instances to outperform decision tree applied on all the features.

On the complete imbalanced dataset, less features, more data instances, and data imbalance led to better predictions for decision tree. On the under-sampled balanced dataset, more features, less data instances, and data balance led better predictions for decision tree.

Perceptron-based neural network obtained an almost perfect score for sensitivity (0.95), confirming again its predictive power in classifying true positive patients. This neural network, however, obtained the worst results on specificity (0.20) among all the methods tried. Compared to the complete imbalanced dataset tests, one rule dropped its general performances score from MCC = +0.27 to MCC = +0.15. Probabilistic neural network obtained again the worst general prediction scores (MCC, accuracy, and F_1_ score) among all the models.

### 4.3 Feature selection

**Fig 4:**
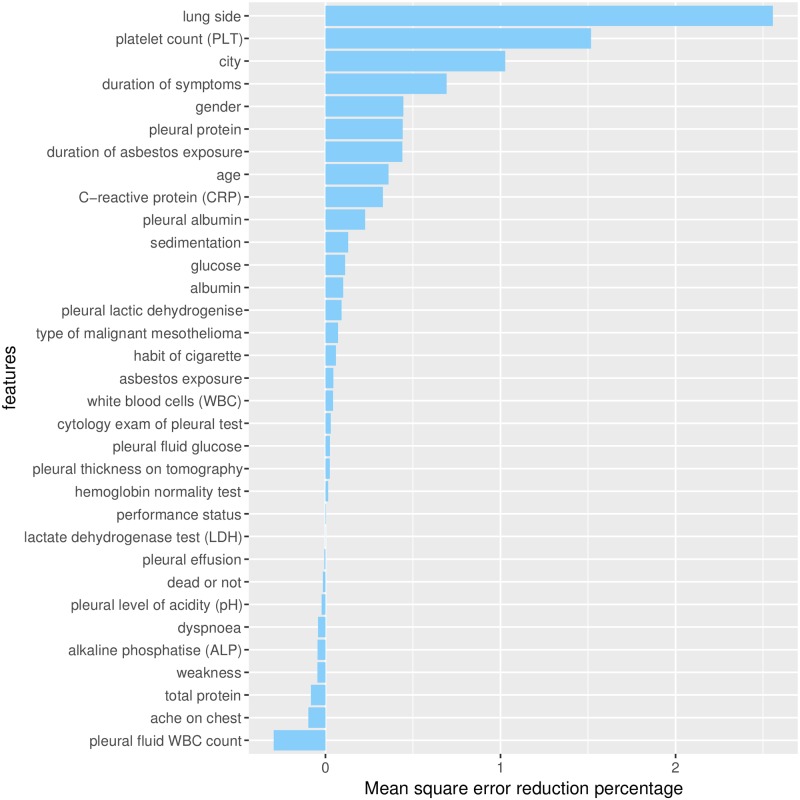
Mean square error (MSE) decrease in accuracy for each feature removal. Random forest feature selection rely on bootstrap aggregation (bagging), and therefore does not have training set, validation set, and test set [69]. The bars represent the drop in the accuracy of the prediction made on the patients’ dataset each time a feature is removed. For each feature, the higher is its accuracy drop when removed, the more important the feature is (Methods).

**Fig 5:**
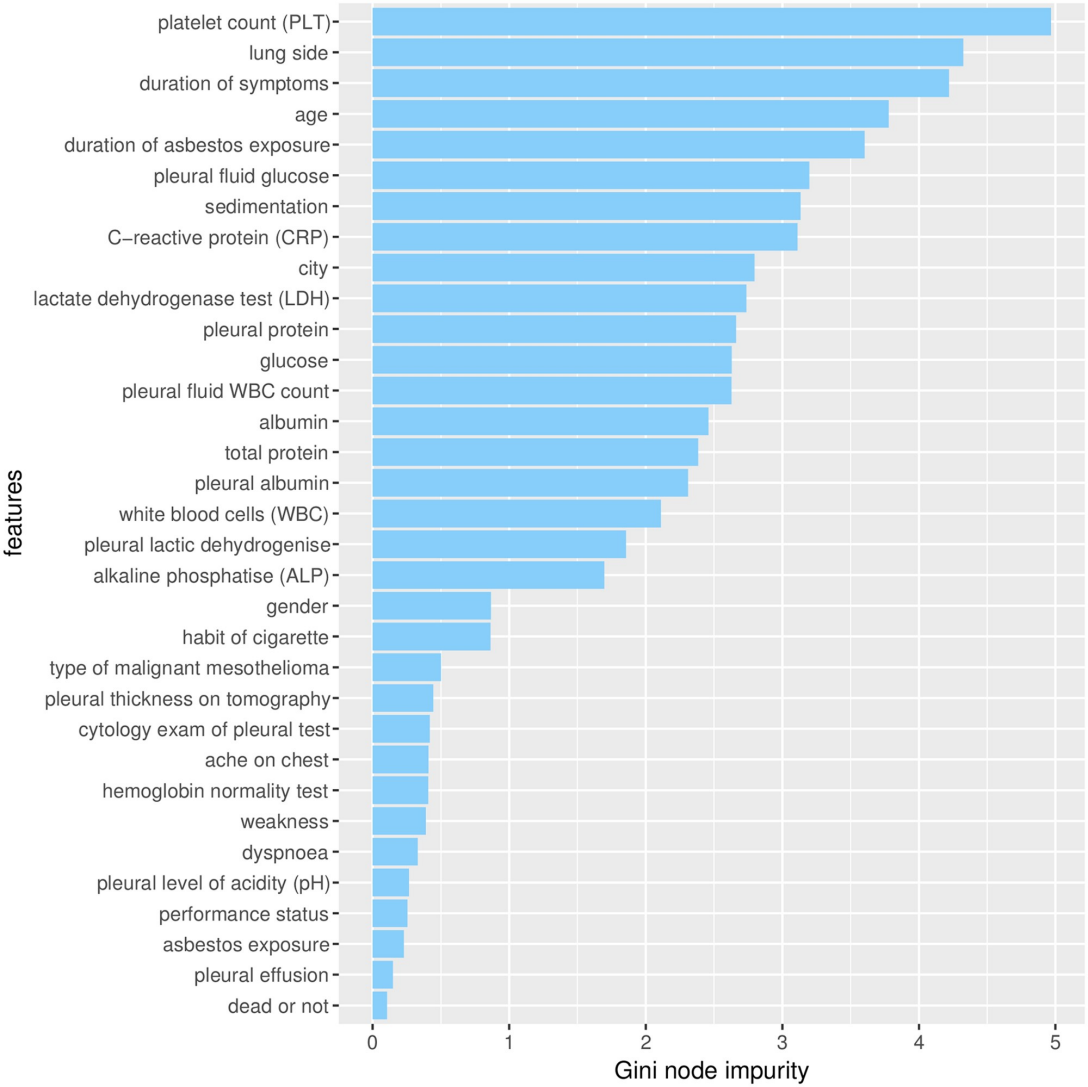
Gini impurity decreases of each random forest tree node. Random forest feature selection rely on bootstrap aggregation (bagging), and therefore does not have training set, validation set, and test set [69]. The bars represent the importance of each feature, measured through the sum of all the Gini impurity index decreases for each specific feature [39] (Methods).

On the feature selection content, the features “lung side” and “platelet count (PLT)” resulted as the most predictive ones among the 33 dataset features (Figs 4 and 5). We measured the importance of each feature with the mean square error decrease (Fig 4) and the Gini node impurity decrease (Fig 5), and these measures highlight “lung side” and “platelet count (PLT)” as the most relevant features for the dataset. In other words, the removal of these two features from the dataset would influence the prediction of the diagnosis more than the removal of the other ones. We selected only “lung side” and “platelet count (PLT)” as top features because they both occupy the first and second positions in both the random forest rankings (Figs 4 and 5).

The merged ranking confirmed the importance of “lung side” and “platelet count (PLT)”, followed by four non-clinical features: “duration of symptoms”, “age”, “city”, “duration of asbestos exposure” (Table 5).

**Table 5:** Merged rank of features. We sorted the features by combining ranking of the node impurity and the ranking of the percentage of MSE decrease in accuracy (Methods).

merged ranking position	feature name	MSE decrease in accuracy %	tree node purity decrease
1	lung side	2.56 × 10^−2^	4.32
2	platelet count (PLT)	1.52 × 10^−2^	4.97
3	duration of symptoms	6.92 × 10^−3^	4.22
4	age	3.60 × 10^−3^	3.78
5	city	1.03 × 10^−2^	2.80
6	duration of asbestos exposure	4.40 × 10^−3^	3.60
7	C-reactive protein (CRP)	3.28 × 10^−3^	3.11
8	pleural protein	4.42 × 10^−3^	2.66
9	sedimentation	1.30 × 10^−3^	3.13
10	glucose	1.12 × 10^−3^	2.63
11	gender	4.45 × 10^−3^	0.87
12	pleural albumin	2.27 × 10^−3^	2.31
13	pleural fluid glucose	2.55 × 10^−4^	3.20
14	albumin	1.01 × 10^−3^	2.46
15	pleural lactic dehydrogenise	9.18 × 10^−4^	1.85
16	lactate dehydrogenase test	3.84 × 10^−6^	2.74
17	white blood cells (WBC)	4.30 × 10^−4^	2.11
18	habit of cigarette	5.92 × 10^−4^	0.86
19	type of malignant mesothelioma	7.23 × 10^−4^	0.50
20	cytology exam of pleural fluid	3.00 × 10^−4^	0.42
21	pleural thickness on tomography	2.49 × 10^−4^	0.44
22	pleural fluid WBC count	−2.96 × 10^−3^	2.63
23	total protein	−8.30 × 10^−4^	2.38
24	alkaline phosphatise (ALP)	−4.54 × 10^−4^	1.70
25	asbestos exposure	4.49 × 10^−4^	0.23
26	hemoglobin normality test	1.54 × 10^−4^	0.41
27	performance status	3.63 × 10^−5^	0.26
28	dyspnoea	−4.23 × 10^−4^	0.33
29	pleural level of acidity (pH)	−2.25 × 10^−4^	0.27
30	ache on chest	−9.76 × 10^−4^	0.41
31	pleural effusion	−6.28 × 10^−5^	0.15
32	weakness	−4.58 × 10^−4^	0.40
33	dead or not	−1.41 × 10^−4^	0.11

The ranking indicated that the less influential features of the predictions are “dead or not”, “weakness”, “pleural effusion” and “ache on chest” (Table 5). Some of these features even have a negative effect on the prediction. “pleural fluid WBC count”, “total protein”, “alkaline phosphatise (ALP)”, “dyspnoea”, “pleural level of acidity (pH)”, “ache on chest”, “pleural effusion”, “weakness”, “dead or not” have negative values in the tree node impurity value list (Fig 4 and MSE accuracy column of Table 5). These features appear not to add useful information, and might even cause overfitting.

The random forest percentage decrease in the Gini node impurity error does not fully confirm this negative effect of the aforementioned features, by for example selecting “pleural fluid WBC count” as the thirteenth most important feature (Fig 5). The difference on the feature selection of these two indexes is caused by their different meaning. The mean square error decrease, in fact, is based upon prediction statistics, while the Gini impurity node decrease is based upon the dataset content information. This meaning difference might lead to such ambiguous cases. Since we want to find the most relevant features of the dataset, and not the least important, we focus on the top features found by both this measures (“lung side” and “platelet count (PLT)”) (Table 5).

### 4.4 Biostatistics analysis

As regression methods are more commonly used to identify variables associated with an outcome (which in this case was presence of mesothelioma among asbestos-exposed individuals), we also performed this traditional statistical modeling technique to allow comparison with our machine learning approaches.

Before regression takes place, it is usual to explore the nature of the relationships between clinical, demographic, radiographic, and laboratory characteristics and the outcome of interest. For this dataset at a significance level of 0.05, patients with mesothelioma were slightly younger (Wilcoxon rank-sum test, *p* = 0.03), more likely to be male (Fisher exact test, *p* = 0.01), were more likely to have mesothelioma in its initial phase (T1 phase in the TNM Classification of Malignant Tumors [70]) (Fisher exact test, *p* = 0.01), and pleural plaques on both lung side (Fisher exact test, *p* = 0.001)(Supplementary section 5). Univariate logistic regression methods (Supplementary section 5) identified age, gender, and lung side as statistically different between cases and controls, when alpha was set at 0.05. CRP levels, duration of symptoms, and duration of asbestos exposure resulted in non-significant trends at an alpha between 0.05 and 0.10. In subsequent multivariate regression analyses, only lung side remained significant.

## 5 Discussion

Our results highlighted several interesting aspects, both regarding the diagnosis prediction and the feature selection. Random forest classifier predicted mesothelioma patients’ diagnosis with high accuracy, both on the complete imbalanced dataset and on the under-sampled balanced dataset. The random forest classifier, in fact, outperformed the probabilistic neural network model previously used to predict the diagnosis of the patients, and all the methods employed in this study. The multi-layer perceptron and one rule outperformed the probabilistic neural network too, but were outperformed by the random forest classifier (Results). These results suggest further usage of random forest and ensemble learning in health informatics.

Our perceptron-based neural network can precisely identify true positive patients having mesothelioma, while our random forest classifier and one rule models can detect true negative patients without mesothelioma with almost perfect specificity. Random forest, in fact, obtained the top prediction results measured with the Matthews correlation coefficient and specificity but, regarding sensitivity, the perceptron resulted in the top performing method with the only sensitivity rate able to predict the majority of true negative elements (both on the complete imbalanced dataset and on the under-sampled balanced dataset). In this scenario, we would suggest biomedical doctors to take advantage of our multi-layer perceptron to predict true positive patients, and to employ our random forest and one rule methods to identify true negative patients.

The presence of pleural plaques on both the lung sides is highly predictive for malignancy. According to our feature selection analysis, “lung side” feature is the most important sign of mesothelioma. If a patient is found to have pleural plaques on both sides of the lung, that patient has a high probability of having a mesothelioma. In fact, the presence of pleural plaques in both sides of the lung as proof of mesothelioma is well known fact in the biomedical community [71]. Doctors consider cancer appearing on both lung sides as a sign of progress in mesothelioma staging, precisely in the advance from stage IIIA to stage IIIB [72]. Also, the association of the “lung side” feature value with the mesothelioma patients’ status confirms the importance of this feature. In this dataset, 22 patients have pleural plaques on both sides (”lung side” value: 2). Among these patients, 16 have mesothelioma in the dataset, meaning the 72.72%. We therefore can see this inference as a correct positive control test for our method.

Low platelet count is strongly related to mesothelioma. According to what our feature selection found, the “platelet count” feature is another influential sign of mesothelioma. Following this indication, we studied the values of this feature and observed that, if patients have a low level of platelet count, they have a high probability of having a mesothelioma.

The “platelet count” feature turned out to be the second most relevant predictive feature of the dataset, ranking second in the list of the features sorted by the mean square error accuracy decrease (Fig 4), first in the list of the features sorted by node impurity (Fig 5) generated by the random forest algorithm, and second in the merged list (Results).

The association of the “platelet count” feature value with the mesothelioma patients’ status confirms the importance of this feature. As mentioned before, the normal range of platelet count for a patient is between 150k and 400k platelets per microliter. The patients having platelet count lower than 150k per microliter in the original dataset are 42. Among these, the mesothelioma ones are 23, that is 54.76% of the total.

We then can state that if a patient has a platelet count value smaller than the lower normality limit (150k platelet per microliter), he/she probably experiences mesothelioma.

**Fig 6:**
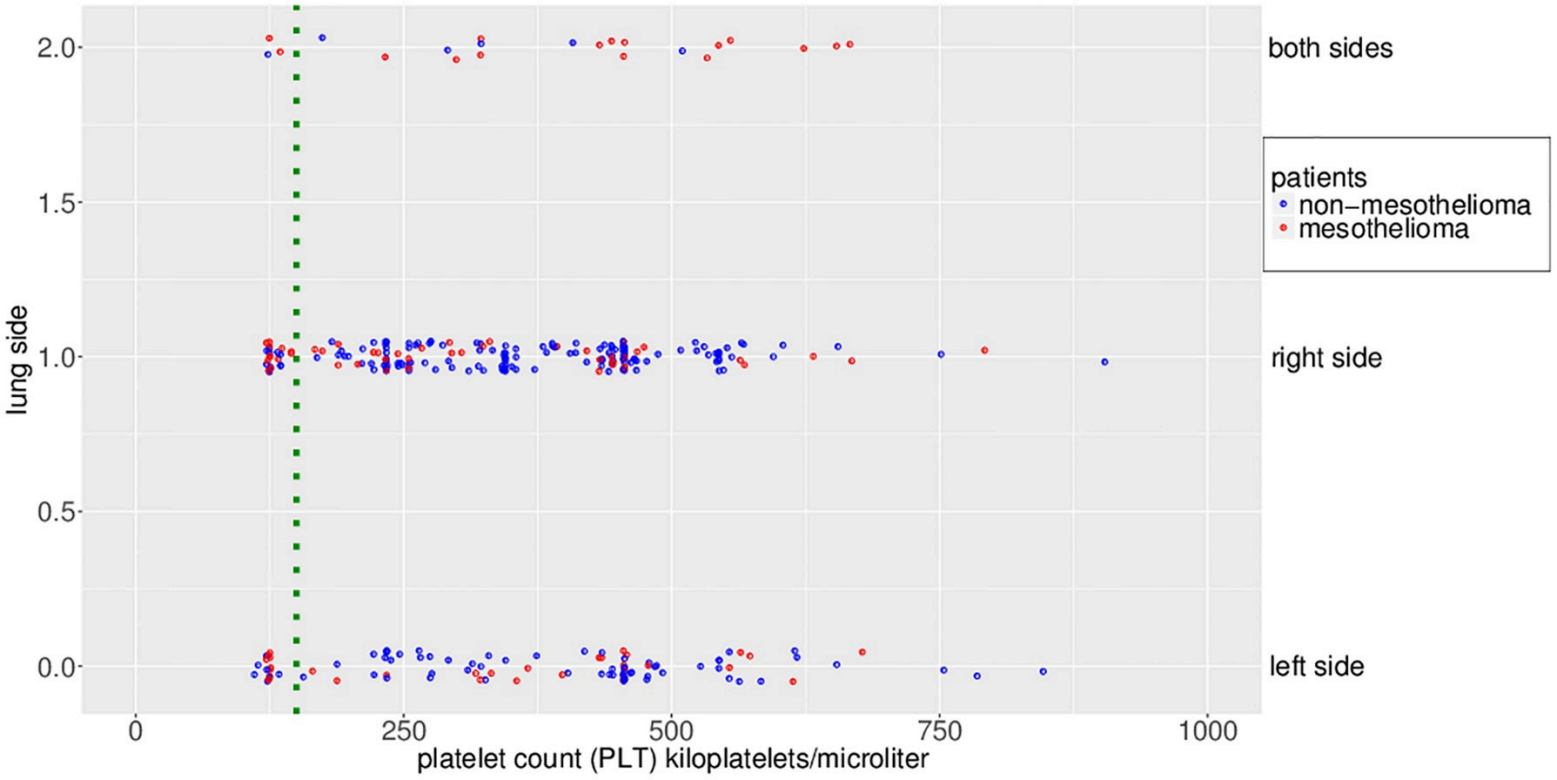
Strip plot of platelet count (PLT) by lung side. We exclude one outlier on the X axis with 3,335 platelet/microliter. Vertical blue dotted line: lower boundary of the platelet count normality test.

In Fig 6, in fact, the majority of dots on the “both sides” horizontal line in the plot are red, meaning that most of these patients have mesothelioma. And also the majority of dots on the left of the green dotted vertical separator (set at the lowest normality limit, 150k platelet per microliter) are red, confirming that the most of patients having platelet count lower than the normality range limit have mesothelioma.

Several research studies (for example, [73]) confirm that low platelet count strongly relates to mesothelioma and it can also happen as a consequence of chemotherapy [74].

The duration of asbestos exposure is an important risk factor, but not among the most important features, according to our random forest feature selection. As we mentioned previously (Introduction), physicians commonly consider the duration of asbestos exposure and the occupational history of the patient as the most relevant risk factors for mesothelioma diagnosis. No information about the occupational history of the dataset patients is available. Regarding “duration of asbestos exposure”, our feature selection model ranked this feature as the sixth most important feature among 33 (Results).

A decision tree applied the two main features selected by random forest (“lung side” and “platelet count”) alone predicted diagnosis of mesothelioma patients with higher accuracy than all the other methods (including decision tree itself) applied to the complete imbalanced dataset.

After having identified the two main features, we trained and tested a classification and regression tree on dataset made only by those two features and by all the 324 patients. Results showed high MCC prediction scores, confirming the importance of “lung side” and “platelet count” in the dataset. These results suggest that physicians could focus on these two features, when analyzing the health record of a patient with signs of mesothelioma, if other feature values were unavailable in his/her medical charts.

Our results about the “lung side” and “platelet count” features can be useful for medical doctors and physicians dealing with patients having mesothelioma symptoms. Our results state that, when analyzing health records of patients having mesothelioma symptoms, physicians should pay more attention to these two highly informative features than to the other features available. Therefore, extra analysis and tests on lung sides and platelet count can be pivotal to diagnose mesothelioma. Additionally, in case only data related to “lung side” and “platelet count” were available for some patients, doctors and biomedical researchers can take advantage of our trained machine learning system to predict their diagnosis. To the best of our knowledge, physicians have not used lung side and platelet count of patients’ health records for mesothelioma diagnosis. The results achieved after applying under-sampling, however, showed that decision tree applied to all the features obtained better prediction scores than decision tree applied only to platelet count and lung side, on the under-sampled balanced dataset. This outcome shows that decision tree applied to the two selected features needs more data instances to outperform decision tree applied to all the features. Decision tree applied to all the features, instead, beats decision tree applied to the two top features, on a perfectly balanced dataset containing the same number of positive data instances and negative data instances.

Additionally, we showed that random forest feature selection provides more insight than standard biostatistics analysis. Random forest, in fact, identified a substantially larger set of important factors that affected mesothelioma risk, when compared to traditional regression methods. Both identified the overwhelming covariate of lung side, but regression methods did not identify platelet count, city, or pleural protein that random forest highlighted.

Regarding the limitations of this study, we have to report that our approach might not generalize well in the mesothelioma context, because of the specificity of the features (for example, the “city” feature, which is the distance from downtown). Our approach, however, can be applied to any patients dataset of any disease available, generate reliable models for diagnosis prediction, and identify the most relevant clinical feature in any of these cases. About feature selection, we have to reaffirm that we built this phase only on random forest, and therefore its results might be biased towards this algorithm (Methods). This limitation might be addressed in the future by employing multiple feature selection methods, and then by comparing and aggregating their results afterwards through advanced correlation rates (such as Spearman’s *ρ* and Kendall *τ* rank correlation coefficients [75], for example).

Under-sampling confirmed its utility to improve the classification results on the minor class (true positives, in our case), even if it brought the limitation of discarding some useful data instances. The under-sampling prediction results, in fact, relate only to 192 patients, and not to the complete dataset made of 324 patients.

Feature work will also include the enhancement of the presented machinery by applying alternative techniques to handle the data class-imbalance [37, 67, 68], the application of our algorithm combination to other disease health record datasets (for example, [41]), the application of alternative machine learning algorithms (for example, latent Dirichlet allocation [76] or probabilistic latent semantic analysis [77]) for the diagnosis prediction, and the possible usage of semantic similarity measures to incorporate similarity information between features (for example, through latent semantic indexing [78]). We also plan to explore the feature dependence in the dataset, to see what feature influence which other features and how.

## Supporting information

S1 File Feature meanings. In this file, we report an accurate description of the meanings of the features.
(PDF)

S1 Fig Barplots of the values of the boolean features. On the left, the patients who do not have mesothelioma; on the right, the mesothelioma patients.
(TIFF)

S2 Fig Barplots of the values of the category features. On the left, the patients who do not have mesothelioma; on the right, the mesothelioma patients.
(TIFF)

S3 Fig Histograms of the values of the time features. On the right, the patients having mesothelioma; on the left, the non-mesothelioma patients.
(TIFF)

S4 Fig Histograms of the real-valued features (part 1/2). On the left, the patients who do not have mesothelioma; on the right, the mesothelioma patients.
(TIFF)

S5 Fig Histograms of the real-valued features (part 2/2). On the left, the patients who do not have mesothelioma; on the right, the mesothelioma patients.
(TIFF)

S1 Table Statistical analysis table summary. Descriptive statistics with median and range for continuous factors and frequencies and percentages for categorical factors.
(TEX)

S2 Table Univariate logistic regression table. We provide descriptive statistics with median and range for continuous factors and frequencies and percentages for categorical factors. NA (not available): not estimable value. reference: reference value used for computing the odd ratios (OR). p-value: statistical p-value for each feature value, computed in relation to the reference value. global p-value: statistical p-value that tests the hypothesis of all the feature values together.
(TEX)

S3 Table Results of the computational predictions of patient diagnosis, including the “diagnosis method” feature in the dataset. (TEX)

S4 Table Results of the computational predictions of patient diagnosis, regarding one rule applied to the two top selected features. The scores are the medians of the the results of ten separate program executions. We applied one rule only to the selected features “lung side” and “platelet count (PLT)”. Dataset imbalance: 29.63% positive data instances (mesothelioma), and 70.37% negative data instances (non-mesothelioma).
(TEX)
