# Supplementary material for: Computational prediction of diagnosis and feature selection on mesothelioma patient health records
Source: PLoS One. 2019 Jan 10;14(1):e0208737. doi: 10.1371/journal.pone.0208737 (PMC6328132; doi:10.1371/journal.pone.0208737)
Supplement: S1 File — In this file, we report an accurate description of the meanings of the features. (PDF) [file pone.0208737.s001.pdf]

Feature meanings

Boolean features

Most of the boolean features are self-evident, and we were able to easily associate false/true meaning labels to the 0/1 values (Table 1). It is possible to understand their meaning from the analysis of the dataset and research studies available online. The only arduous task was to discriminate to the boolean features from the category features having binary values whose meaning is not true and false (for example, “gender”, which clearly is male or female).

Following the scientific literature tradition in computer science, we assigned the value 0 to “false” and the value 1 to “true” for all the boolean features listed hereafter.

The boolean feature named “**ache on chest**” is related to the presence or absence in patient of pain in the abdomen area and in particular in chest in case of pleural mesothelioma.

The boolean feature named “**asbestos exposure**” refers to whether or not a patient has been exposed to the asbestos during his/her life.

A “**cytology exam of pleural fluid**” is a laboratory test to detect cancer cells and certain other cells in the area that surrounds the lungs, the pleural space. In a normal result normal cells are seen; in an abnormal test, there are cancerous (malignant) cells and this may mean there is a cancerous tumor [79].

“**Dead or not**” refers to whether or not a patient is still alive.

The feature named “**diagnosis method**” has possible values are 0 and 1. Its values are exactly the same of the “class of diagnosis” target column of the dataset, which state if each row is related to a non-mesothelioma patient (value 0), or to a patient having a mesothelioma (value 1). This feature states if the patient has had a mesothelioma diagnosed by a common diagnosis method, for example Blood tests such as MESOMARK and SOMAmer, Imaging tests such as MRIs, CT scans, PET scans, X-rays, Biopsies to investigate cancerous growth in tissue samples [80].

Another boolean feature is “**dyspnoea**”, which means shortness of breath and refers to whether a patient has difficulty breathing.

“**Hemoglobin normality test**” refers to the hemoglobin test that measures how much hemoglobin is in blood. Normal results for adults vary, but in general are between 14-18 gm/dl for men, and 12-16 gm/dl for women. Higher hemoglobin level is most often caused by low oxygen levels in the blood (hypoxia), present over a long period of time, due to lung diseases [81].

In some cases, the presence of an “**pleural effusion**” signals advancement of the disease or mesothelioma. With mesothelioma, an effusion is a common symptom that can inhibit the normal function of the affected organ [82].

“**Pleural level of acidity**” means whether or not the pleural fluid is lower than the normal pleural fluid, which has a pH around 7.60–7.64. Metabolic activity within the pleural space results in a low pleural pH (< 7.3) in the several situations, for example complicated parapneumonic effusions and emphysema, malignancy, tuberculous pleuritis, oesophageal rupture, rheumatoid pleuritis and lupus pleuritis [83].

“**Pleural thickness on tomography**” is a descriptive term given to describe any form of thickening involving either the parietal or visceral pleura. Pleural thickening is due to tumor infiltration: it could occur with both pleural plaques and with pleural disease [84, 85].

“**Weakness**” or asthenia refers to whether or not patients feel lack of strength.

Category features

"**City**" refers to the place of provenance of the patients (Table 1). The values refer to the place where the patients used to live related to the city downtown. The value 0 means that the patient used to live in the city center, the value 1 refers to the city center surroundings, and so on. Finally, the value 8 means maximum distance from the city downtown among the patients locations.

The patients were divided in two groups based on "**gender**", indeed with occupational asbestos exposure, pleural mesotheliomas usually occur in a male; women are less likely to work in contaminated areas [86]. According to the original dataset curators, 1 means men and 0 means women.

The feature named "**habit of cigarette**" is characterized by four category based upon patient's habit of smoking, where 0 means non-smoker, 1 means rare smoker, 2 means regular smoker, and 3 means frequent smoker.

For "keep side", the possible values are 0, 1 and 2. This feature is related to the side of the lung which has pleural plaques or clinical signs similar to mesothelioma traces, with 0 referring to the left side of the lung, 1 referring to the right side of the lung, and 2 referring to both the lung sides. If the patient does not have mesothelioma, it refers to the presence of pleural plaques in the lung. We then changed the name of this feature to the more appropriate "**lung side**".

Patient "**performance status**" plays a role both in shaping prognosis and in determining the best treatment for a patient with cancer. The performance status is a feature characterized by two category and estimates whether or not patients are able to perform certain activities of daily living [87]. The value 0 means able to perform some everyday tasks, while 1 means mesothelioma and unable to perform some everyday tasks.

The feature named "**type of malignant mesothelioma**" refers to mesothelioma staging to which the patient's symptoms seem to belong, according to the TNM Classification of Malignant Tumors [**?**, 88]. For the patients having mesothelioma, this feature value means that their tumor can be classified in one of the TNM categories. For patients who do not have mesothelioma, this feature value means that their pleural plaques and general symptoms could be associated to one of the TNM categories. The values are: 0 meaning no evidence of a primary tumor (T0 phase in the TNM classification) or initial phase of the disease (T1 phase in the TNM classification); 1 meaning middle phase of the disease (T2 and T3 phases in the TNM classification); 2 meaning last and advanced phase of the disease (T4 phase in the TNM classification).

Time features

The dataset has only three time features, all measured in years. The numerical "**age**" of the samples goes from 19 to 85 years.

One of the most important hallmarks to take into account is the "**duration of asbestos exposure**". Asbestos exposure is the leading cause for mesothelioma [89–91].

"**Duration of symptoms**" refers to the time period, in years, in which the patients show symptoms. Generally, those who develop asbestos-related diseases show no signs of illness for a long time after their first exposure. It can take from 10 to 40 years or more for symptoms of an asbestos-related condition to appear [92].

Real valued features

The remaining features of the dataset had real values, and therefore were the easiest to interpret. Most of them report values of standard clinical tests, which were set to be in specific value ranges. This helped us in the comprehension of each feature meaning.

The normal range for "**albumin**" is 3.4 to 5.4 g/dL a lower-than-normal level of blood albumin may be a sign of many diseases such as liver and kidney disease. People with the lowest levels of the blood protein albumin are less likely to live beyond a year with pleural mesothelioma. That is the finding of Yao et al. who stated this abundant protein might offer one of the simplest ways to predict mesothelioma prognosis [93]. The feature "**albumin**" should not be confused with "pleural albumin", that is another real-valued feature in this dataset.

"**Alkaline phosphatase (ALP)**" is a protein found in all body tissues and the normal range is 44 to 147 IU/L. The alkaline phosphatase test is used to help detect liver disease or bone disorders. Any condition that affects the liver, or bone growth, causes increased activity of bone cells, can affect ALP levels in the blood. An ALP test may be used, for example, to detect cancers that have spread to the bones [94].

"**Lactate dehydrogenase test (LDH)**" is a protein that helps produce energy in the body. An LDH test measures the amount of LDH in the blood and the normal value range is 105 to 333 IU/L. LDH is found in many body tissues such as the heart, liver, kidney, skeletal muscle, brain, blood cells, and lungs. High LDH were found to be prognostic indicators in mesothelioma. LDH-3 is highest in the lung [95, 96].

"**C-reactive protein (CRP)**", an acute phase reactant, has been noted to be significantly elevated in patients with metastatic disease across a variety of solid organ and hematological malignancies, including pleural mesothelioma (MPM) [97, 98]. In a retrospective study of 115 patients with a pathologically confirmed diagnosis of pleural mesothelioma, elevated C-reactive protein ($\geq$1 mg/dL) was shown to be an independent indicator of poor prognosis (HR=2.07; 95% CI: 1.23-3.46; P=0.001) [99, 100].

Another standard laboratory test is "**glucose**", that is a blood glucose test which measures the amount of glucose in a sample of blood. A level between 70 and 100 mg/dL is considered normal and higher value is linked to a non-healthy condition of the patient [81].

Another important feature is the originally named "white blood" and refers to the count of white blood cells (leukocytes) in the pleural fluid. Normal pleural fluid has fewer than 1000 white blood cells (WBCs) per mcL. The measurements of pleural fluid WBC count is helpful in the diagnosis and management of patients with pleural effusion. In general, a WBC count greater than 1,000 cells/microliter suggests an exudate, while most transudates have WBC counts of lower than 1,000 cells/microliter. WBC counts greater than 10,000 cells/microliter could be related to malignancy [101]. For these reasons, we changed the name of this feature to "**pleural fluid WBC count**".

A "**platelet count (PLT)**" is a lab test to measure how many platelets you have in your blood. The normal number of platelets in the blood is 150,000 to 400,000 platelets per microliter (mcL) [102] and a value greater than 400,000 mcL is considered as a poor prognostic factors [82]. Pleural fluid is drawn out of the pleural space in a process called thoracentesis, the fluid is analyzed and some values can be considered as prognostic factors.

"**Pleural albumin**" (not to be confused with "albumin", that is another real-valued feature in this dataset) is the level of albumin in the pleural fluid and if the difference between the albumin level in the blood and the pleural fluid is greater than 1.2 g/dL, this suggests that the patient has a transudative pleural effusion [103].

A low level of "**pleural fluid glucose**" can be linked to infection or malignancy [103]. The upper limit of the normal "**pleural lactic dehydrogenase**" is 200 IU/L. A high lactic dehydrogenase indicates that pericardial fluid, peritoneal or pleural fluid is an exudate, while a low level indicates it is transudate.

Normal "**pleural proteins**" count is less than 1-2 g/dL. Pleural effusions are classified as transudates or exudates on the basis of the fluid protein level, classically, a pleural fluid protein level greater than 30 g/L is an exudate and lower than 30 g/L is a

transudate, in the context of a normal serum protein level [83].

The "**sedimentation rate**" (sed rate) blood test measures how quickly red blood cells (erythrocytes) settle in a test tube in one hour (mm/hr). The more red cells that fall to the bottom of the test tube in one hour, the higher the sedimentation rate, the normal rate is 15-30 mm/hr. Blood tests, in patients with mesothelioma, can reveal an elevated erythrocyte sedimentation rate (ESR) [104], and there have been isolated case reports of mesothelioma associated with autoimmune hemolytic anemia [105].

"**Total protein**", also known as serum total protein, is a biochemical test for measuring the total amount of protein in serum. The reference range for total protein is typically 6.0–8.0 g/dl [106]. Concentrations below the reference range usually reflect low albumin concentration and may refers to liver disorder and kidney disorder. Elevated total protein may indicate: inflammation or infections, such as viral hepatitis B or C, or HIV and bone marrow disorders [107, 108].

Standard laboratory values including white blood "cell count (WBC)" is the prognostic factors for mesothelioma [109]. This test measures the number and quality of white blood cells, white cells count can detect hidden infections and undiagnosed medical conditions. The normal number of white blood cells in the blood is 4,500 to 11,000 white blood cells per microliter, and people with mesothelioma can have an high white blood cell level (leukocytosis) [110]. We changed the name of this feature into "**white blood cells (WBC)**".

After investigating the meanings of all these features, we contacted via email one of the curators of the original dataset (Orham Er, [**?**]), who confirmed all our findings.

References

79. Pfenninger JL, Fowler GC. Pfenninger and Fowler's procedures for primary care: expert consult. Elsevier Health Sciences; 2010.

80. Asbestos. Mesothelioma diagnosis;. `https://www.asbestos.com/mesothelioma/diagnosis.php`. URL visited on 31st October 2016.

81. Goljan EF. Rapid review pathology: with student consult online access. Elsevier Health Sciences; 2013.

82. Moore AJ, Parker RJ, Wiggins J. Malignant mesothelioma. Orphanet Journal of Rare Diseases. 2008;3(1):1.

83. Rahman NM, Chapman SJ, Davies RJ. Pleural effusion: a structured approach to care. British Medical Bulletin. 2004;72(1):31–47.

84. Lange S, Stark P. Radiology of chest diseases. Thieme, Stuttgart, Germany; 1990.

85. Downer NJ, Ali NJ, Au-Yong IT. Investigating pleural thickening. British Medical Journal (BMJ). 2013; p. 1–5.

86. Metintas M, Metintas S, Ak G, Erginel S, Alatas F, Kurt E, et al. Epidemiology of pleural mesothelioma in a population with non-occupational asbestos exposure. Respirology. 2008;13(1):117–121.

87. West HJ, Jin JO. Performance status in patients with cancer. Journal of American Medical Association (JAMA) Oncology. 2015;1(7):998–998.

88. Brierley JD, Gospodarowicz MK, Wittekind C. TNM classification of malignant tumours. John Wiley & Sons; 2016.

89. Yazicioglu S, Ilcayto R, Balci K, Sayli B, Yorulmaz B. Pleural calcification, pleural mesotheliomas, and bronchial cancers caused by tremolite dust. Thorax. 1980;35(8):564–569.

90. Selikoff IJ, Churg J, Hammond EC. Relation between exposure to asbestos and mesothelioma. New England Journal of Medicine. 1965;272(11):560–565.

91. Wagner J, Sleggs C, Marchand P. Diffuse pleural mesothelioma and asbestos exposure in the North Western Cape Province. British Journal of Industrial Medicine. 1960;17(4):260–271.

92. Centers for Disease Control and Prevention. Agency for toxic substances and disease registry 2009. CDC/ATSDR Strategic Plan for Public Health Workforce Development Executive Summary. 2012;.

93. Yao ZH, Tian GY, Yang SX, Wan YY, Kang YM, Liu QH, et al. Serum albumin as a significant prognostic factor in patients with malignant pleural mesothelioma. Tumor Biology. 2014;35(7):6839–6845.

94. Martin P. Approach to the patient with liver disease. In: Elsevier Inc.; 2011.

95. Pass HI. Biomarkers and prognostic factors for mesothelioma. Annals of Cardiothoracic Surgery. 2012;1(4):449–456.

96. McPherson RA, Pincus MR. Henry's clinical diagnosis and management by laboratory methods. Elsevier Health Sciences; 2016.

97. Weinstein PS, Skinner M, Sipe JD, Lokich JJ, Zamcheck N, Cohen AS. Acute-phase proteins or tumour markers: the role of SAA, SAP, CRP and CEA as indicators of metastasis in a broad spectrum of neoplastic diseases. Scandinavian Journal of Immunology. 1984;19(3):193–198.

98. Roxburgh CS, McMillan DC. Role of systemic inflammatory response in predicting survival in patients with primary operable cancer. Future Oncology. 2010;6(1):149–163.

99. Ghanim B, Hoda MA, Winter MP, Klikovits T, Alimohammadi A, Hegedus B, et al. Pretreatment serum C-reactive protein levels predict benefit from multimodality treatment including radical surgery in malignant pleural mesothelioma: a retrospective multicenter analysis. Annals of Surgery. 2012;256(2):357–362.

100. Linton A, van Zandwijk N, Reid G, Clarke S, Cao C, Kao S. Inflammation in malignant mesothelioma: friend or foe? Annals of Cardiothoracic Surgery. 2012;1(4):516.

101. Burt BM, Rodig SJ, Tilleman TR, Elbardissi AW, Bueno R, Sugarbaker DJ. Circulating and tumor-infiltrating myeloid cells predict survival in human pleural mesothelioma. Cancer. 2011;117(22):5234–5244.

102. Hoffman R, Silberstein LE, Heslop H, Weitz J. Hematology: basic principles and practice. Elsevier Health Sciences; 2013.

103. Life In The Fast Lane. Pleural fluid analysis; 2015. `http://lifeinthefastlane.com/investigations/pleural-fluid-analysis/`. URL visited on 31st October 2016.

104. Elmes PC, Simpson MJ. The clinical aspects of mesothelioma. QJM: an International Journal of Medicine. 1976;45(3):427–449.

105. Selleslag DL, Geraghty RJ, Ganesan TS, Slevin ML, Wrigley PF, Brown R. Autoimmune haemolytic anaemia associated with malignant peritoneal mesothelioma. Acta Clinica Belgica. 1989;44(3):199–201.

106. Pathology Harmony. Harmonisation of reference intervals; 2013.

107. Mayo Clinic Staff. High blood protein: causes; 2016. http://www.mayoclinic.org/symptoms/high-blood-protein/basics/causes/sym-20050599. URL visited on 31st October 2016.

108. Lab Tests Online. Total protein and A/G ratio; 2016. http://labtestsonline.org/understanding/analytes/tp/tab/test. URL visited on 31st October 2016.

109. Pass HI. Biomarkers and prognostic factors for mesothelioma. Annals of Cardiothoracic Surgery. 2012;1(4):449–456.

110. Dollinger M, Tempero M, Mulvihill S. Everyone's guide to cancer therapy: how cancer is diagnosed, treated, and managed day to day. Andrews McMeel Publishing; 2002.
