# Supplementary figures and images for: Computational prediction of diagnosis and feature selection on mesothelioma patient health records
Source: PLoS One. 2019 Jan 10;14(1):e0208737. doi: 10.1371/journal.pone.0208737 (PMC6328132; doi:10.1371/journal.pone.0208737)

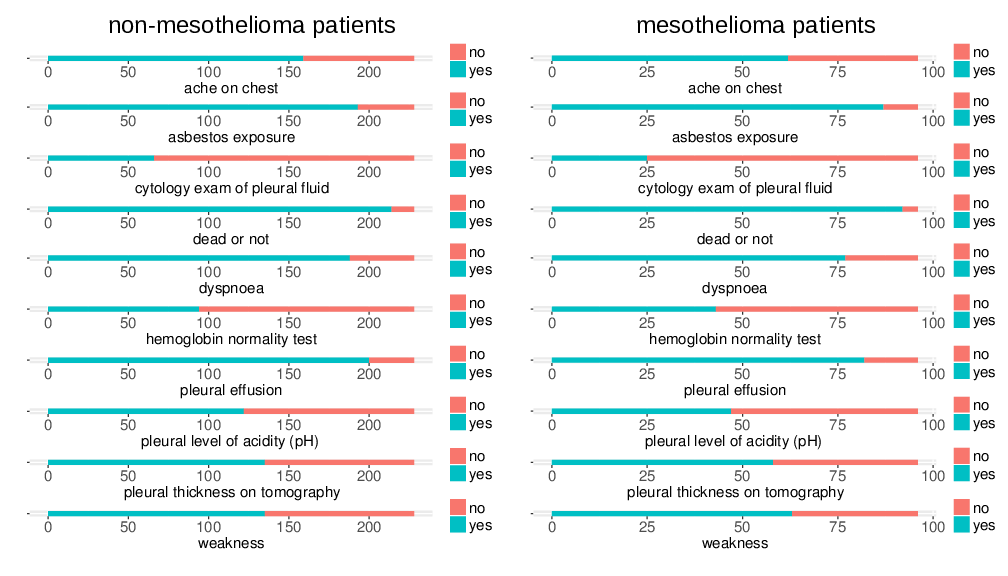

Supplement: S1 Fig — On the left, the patients who do not have mesothelioma; on the right, the mesothelioma patients. (TIFF) [file pone.0208737.s002.tiff]

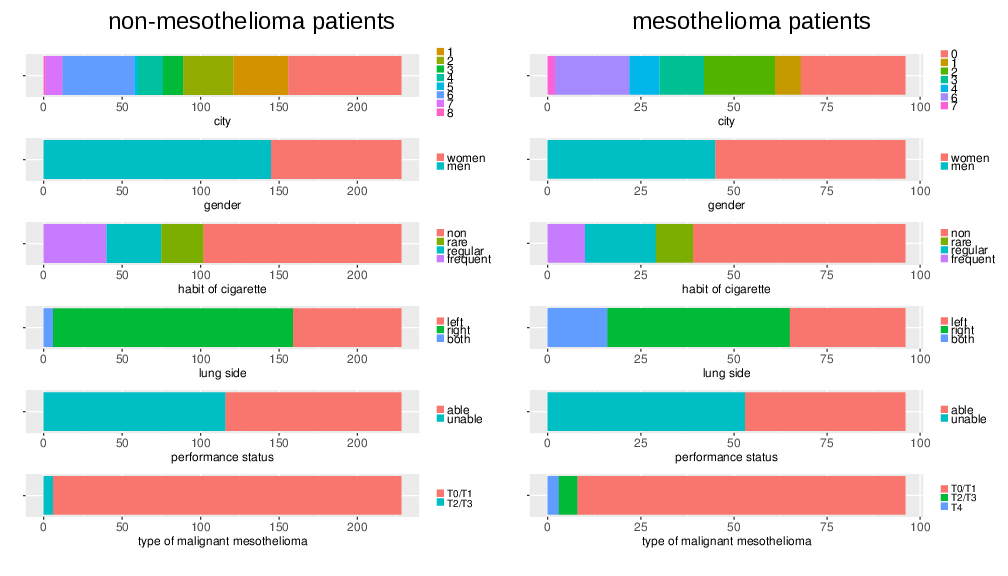

Supplement: S2 Fig — On the left, the patients who do not have mesothelioma; on the right, the mesothelioma patients. (TIFF) [file pone.0208737.s003.tiff]

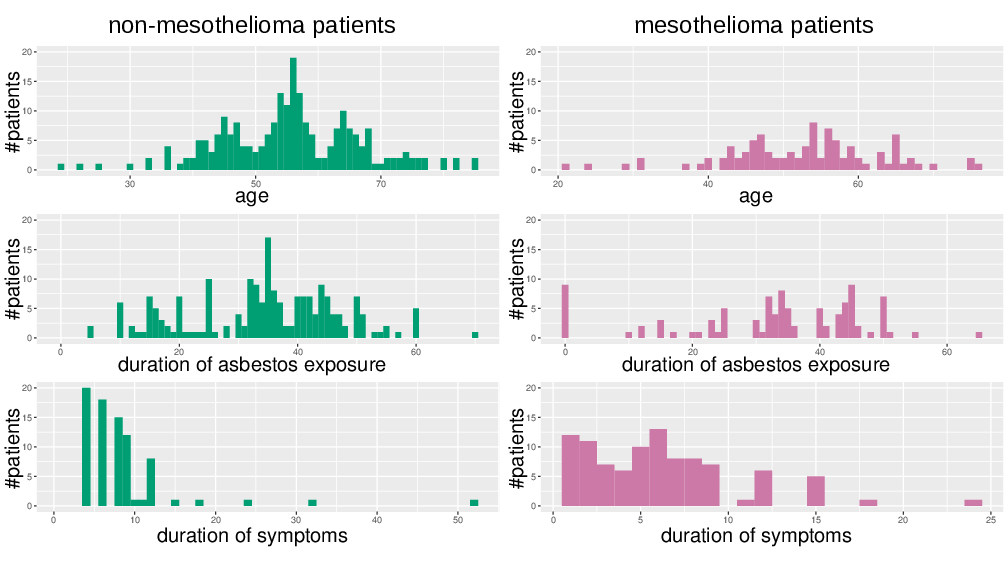

Supplement: S3 Fig — On the right, the patients having mesothelioma; on the left, the non-mesothelioma patients. (TIFF) [file pone.0208737.s004.tiff]

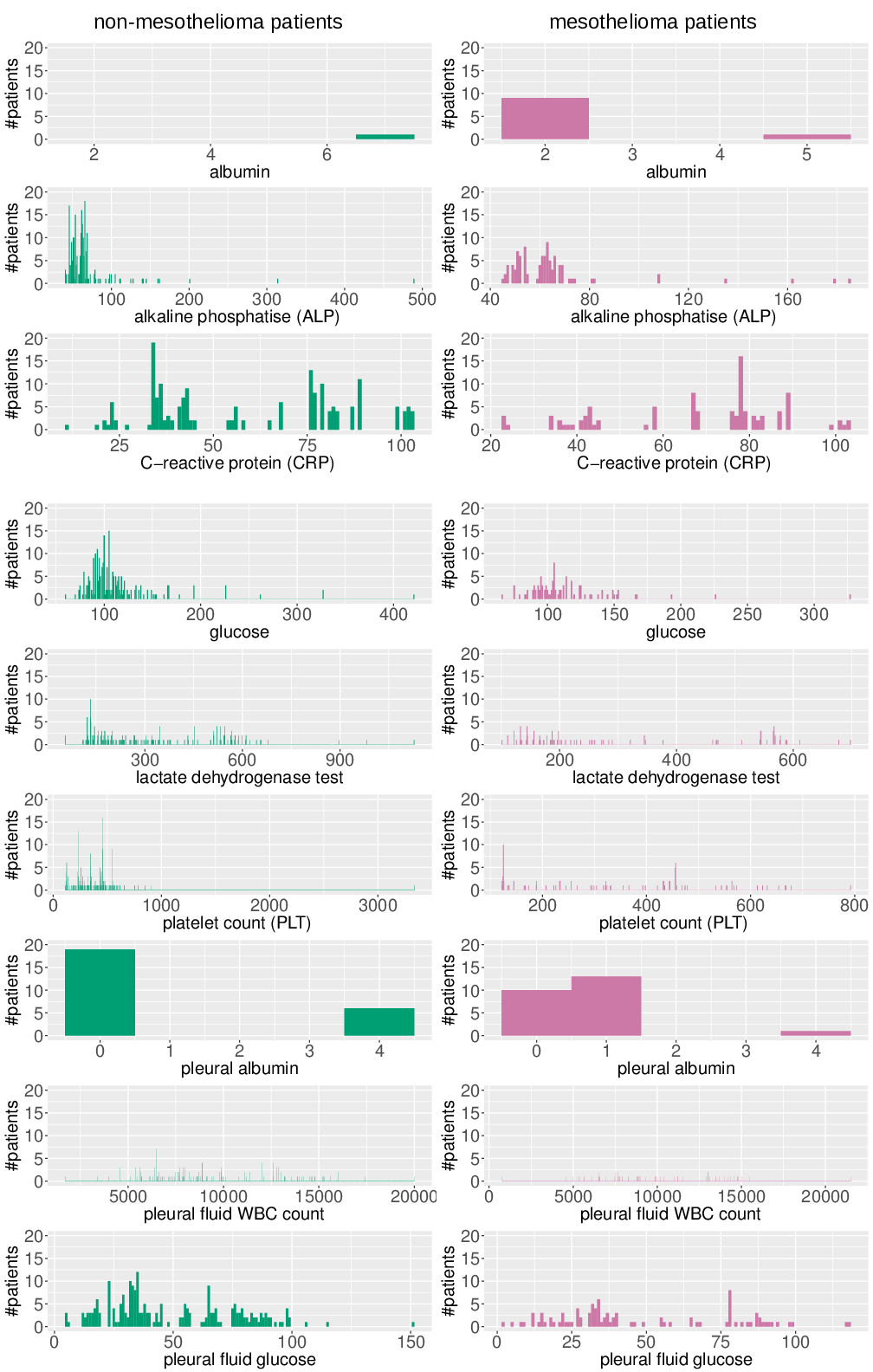

Supplement: S4 Fig — On the left, the patients who do not have mesothelioma; on the right, the mesothelioma patients. (TIFF) [file pone.0208737.s005.tiff]

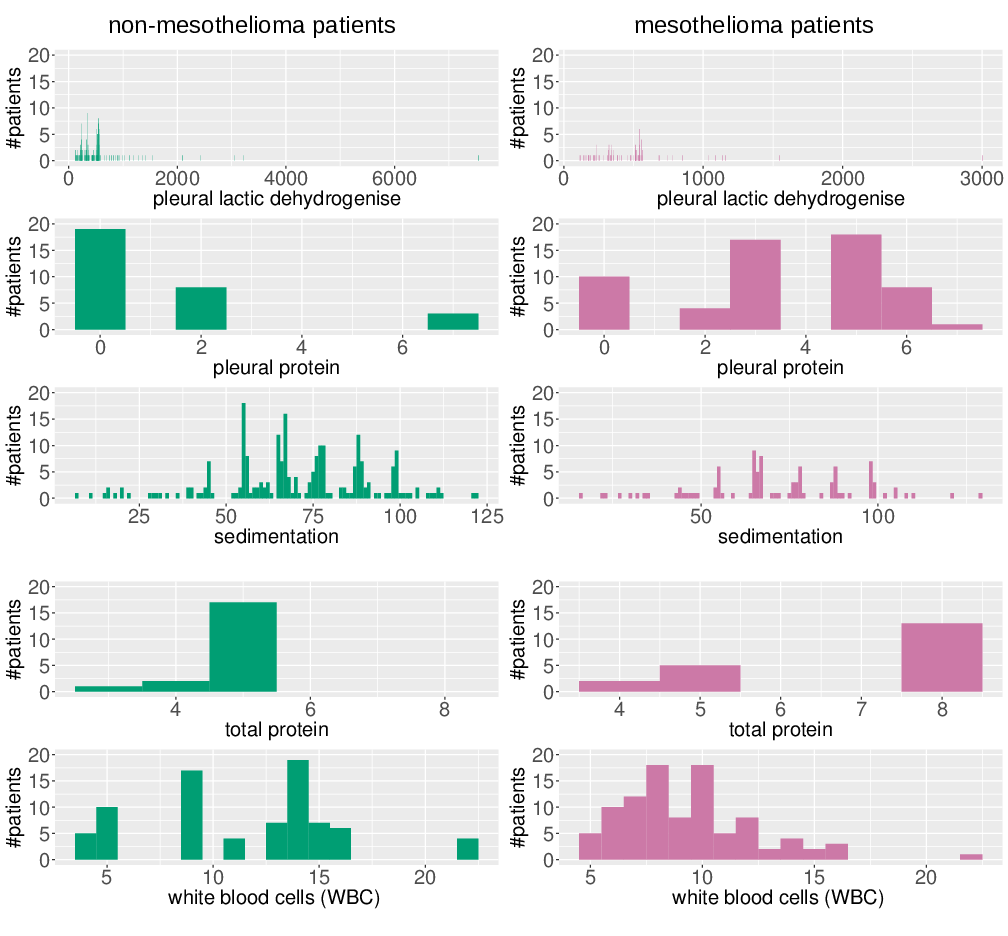

Supplement: S5 Fig — On the left, the patients who do not have mesothelioma; on the right, the mesothelioma patients. (TIFF) [file pone.0208737.s006.tiff]
